# VirDetector: a bioinformatic pipeline for virus surveillance using nanopore sequencing

**DOI:** 10.1093/bioinformatics/btaf029

**Published:** 2025-01-21

**Authors:** Nick Laurenz Kaiser, Martin H Groschup, Balal Sadeghi

**Affiliations:** Federal Research Institute for Animal Health, Institute of Novel and Emerging Infectious Diseases, Friedrich-Loeffler-Institut, Greifswald, Insel Riems 17493, Germany; Federal Research Institute for Animal Health, Institute of Novel and Emerging Infectious Diseases, Friedrich-Loeffler-Institut, Greifswald, Insel Riems 17493, Germany; Federal Research Institute for Animal Health, Institute of Novel and Emerging Infectious Diseases, Friedrich-Loeffler-Institut, Greifswald, Insel Riems 17493, Germany

## Abstract

**Summary:**

Virus surveillance programmes are designed to counter the growing threat of viral outbreaks to human health. Nanopore sequencing, in particular, has proven to be suitable for this purpose, as it is readily available and provides rapid results. However, as special bioinformatic programs are required to extract the relevant information from the sequencing data, applications are needed that allow users without extensive bioinformatics knowledge to carry out the relevant analysis steps. We present VirDetector, a bioinformatic pipeline for virus surveillance using nanopore sequencing. The pipeline automatically installs all required programs and databases and allows all its steps to be executed with a single console command. After preprocessing the samples, including the possibility for basecalling, the pipeline classifies each sample taxonomically and reconstructs the viral consensus genomes, which are then used in phylogenetic analyses. This streamlined workflow provides a user-friendly and efficient solution for monitoring viral pathogens.

**Availability and implementation:**

VirDetector is freely available at https://github.com/NLKaiser/VirDetector and https://zenodo.org/records/14637302 (10.5281/zenodo.14637302).

## 1 Introduction

There are many viruses that pose a threat to the well-being of humans and animals. While the Ebola virus harms local communities, we have experienced the consequences of a global outbreak with the coronavirus pandemic. The economic damage caused by viruses results, e.g. from the overburdening of healthcare systems or an outbreak in livestock herds often affecting small-scale farmers. Viruses which can be transmitted between humans and animals, such as the Rift Valley fever virus, are referred to as zoonoses. Mosquitoes or ticks often serve as vectors for these zoonotic viruses. Highly pathogenic viruses, assigned to biosafety risk groups 3 and 4, are particularly hazardous for people in resource-poor regions. Infections are frequently underdiagnosed and neither vaccines nor therapies are available. As a change in the global climate is likely to cause these viruses to spread to previously unexposed areas in the future, the incursion risk is steadily increasing for developed countries in the northern hemisphere as well.

Virus surveillance programmes counteract these threats by detecting outbreaks early, enabling decision-makers and stakeholders to take informed action at an early stage. Nanopore sequencing has proven to be particularly suitable for this task due to comparatively low requirements for sample preparation. Portable sequencers also enable programmes in remote regions where the use of other measures is often impractical due to limited financial resources. Successful implementation of nanopore sequencing has already been demonstrated both in clinical settings, e.g. for the influenza A virus, and in field trials, such as for Ebola and several other viruses (Hoenen *et al.* 2016, Williams *et al.* 2023).

To transform the raw output of nanopore sequencing into nucleotide sequences, the data is first basecalled. The resulting reads are then usually filtered based on quality and length. To determine the characteristics of a virus, the viral genome can be reconstructed. This can be done by mapping the remaining reads against a reference sequence and then assembling them. The tools used for these steps are specifically designed for the long, error-prone reads that are generated by nanopore sequencing. As running all the required programs manually would be very time-consuming and labour-intensive, it is common practice to combine them within a pipeline using workflow management systems. This allows the automatic sequential execution of all steps so that the user receives all relevant results with a single program call. Another advantage of bioinformatic pipelines is that the results they produce are easily reproducible, thus increasing standardisation and quality assurance in sequence analysis.

We present VirDetector, a bioinformatic pipeline for virus surveillance using nanopore sequencing. It is designed to automate routine bioinformatic analyses in scenarios where samples contain a single virus of interest. It enables the rapid detection and characterisation of viruses during viral outbreaks, whether they impact humans or animals, allowing decision makers to take timely and informed action. In addition, VirDetector can be used to standardise the analysis of sequencing data in research settings. During the development of the pipeline, special care was taken to ensure that its use requires only a basic understanding of bioinformatics, making it accessible to a wide range of users. All required dependencies are installed automatically and all steps can be executed directly afterwards with a single console command. VirDetector can process both the raw data to be basecalled and FASTQ files as input. In a metagenomics step, each sample is classified taxonomically. This can help to determine if any viruses other than the one of interest, such as contaminants, are present in the sample. Both a reference-based approach and a hybrid approach are available to reconstruct the viral genome. Afterwards, structural variants are identified and phylogenetic analyses are carried out. The programs used within the pipeline as well as the databases used can be easily adapted via a config file. VirDetector can therefore be used in a wide variety of virus monitoring settings using nanopore sequencing.

## 2 Materials and methods

**Figure 1.**
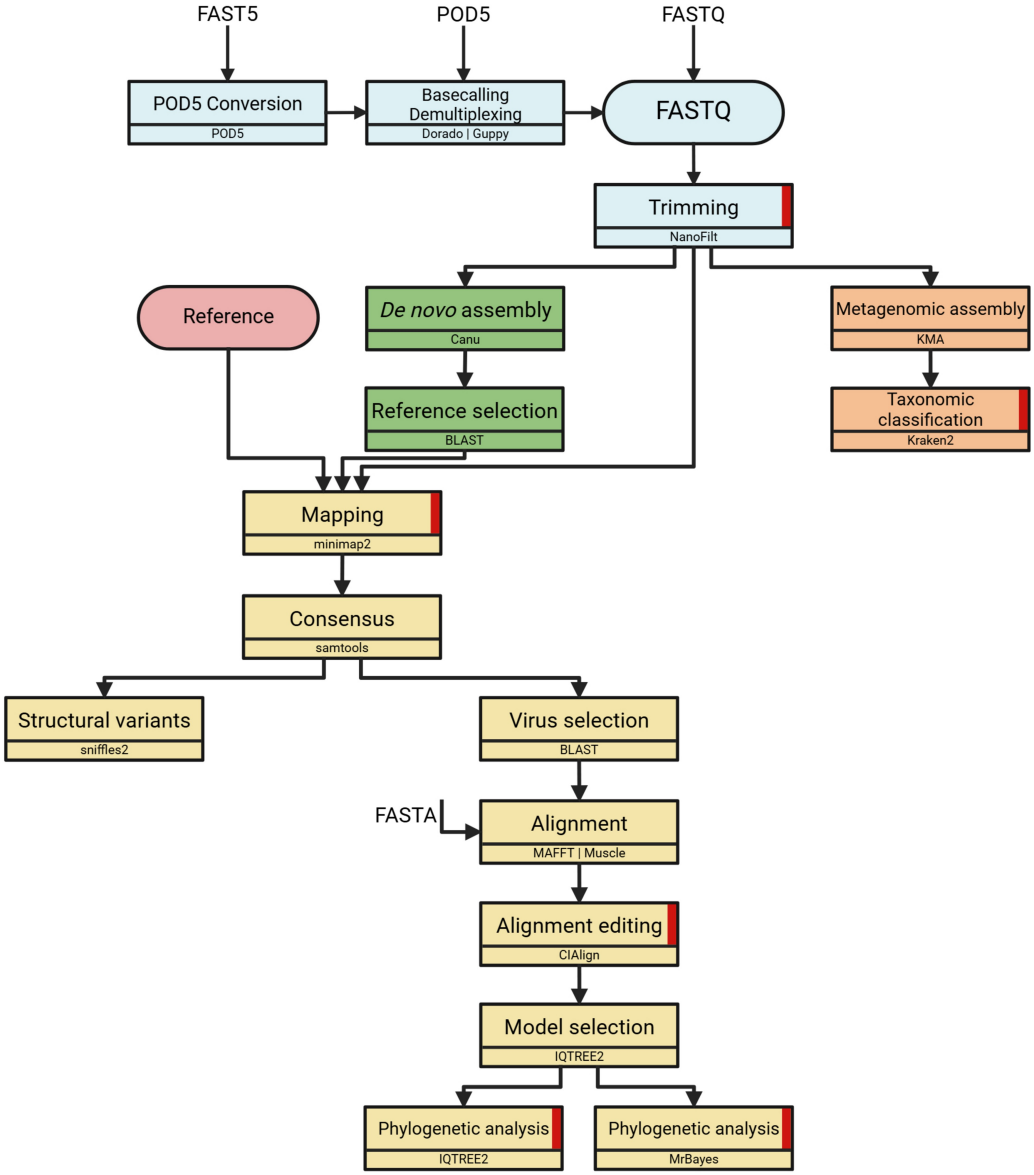
The VirDetector workflow. Indicated on the arrows are the different file types that the user can provide via the input folder. The colouring of the fields corresponds to the individual steps of the pipeline, the preprocessing of the samples, a reference-based assembly, a hybrid assembly and metagenomic classification. All steps that have a graphical output are marked with red. Created in BioRender. Kaiser, N. (2024) https://BioRender.com/u19y358.

VirDetector can be installed either via the Conda package manager or via Docker (https://docs.anaconda.com/, Merkel 2014). The installation of all components and the download of all databases is done automatically. The pipeline is implemented using Nextflow (Supplementary Table S1) (Di Tommaso *et al.* 2017). Figure 1 shows the workflow of the VirDetector pipeline.

### 2.1 Preprocessing

In the first preprocessing step, all files specified in the input folder are processed based on their file extension. The pipeline accepts FAST5, POD5, and FASTQ files, zipped or unzipped, as input. FAST5 files are automatically converted to POD5 format which enables faster basecalling (https://github.com/nanoporetech/pod5-file-format). Files within the input folder itself are considered as separate samples, those within subfolders are merged into one. For basecalling, the user can choose between Dorado, if a GPU is available, and the CPU version of Guppy (https://github.com/nanoporetech/dorado, https://nanoporetech.com/). Since Dorado can achieve more accurate results, we recommend using it here. Demultiplexing of the results is optionally available. Each sample is then filtered using NanoFilt (De Coster *et al.* 2018). Quality plots are created with NanoComp to compare the unfiltered and filtered reads (De Coster and Rademakers 2023).

### 2.2 Genome reconstruction

The viral genome can either be assembled by using a reference sequence or in a hybrid approach. In the hybrid approach, the reads are first assembled *de novo* using Canu (Koren *et al.* 2017). The resulting contigs are then blasted against the given database and a reference sequence is selected based on the highest bit-score (Camacho *et al.* 2009). The subsequent steps are identical to the reference-based assembly. The reads of each sample are mapped against the reference using minimap2 (Li 2021). Qualimap reports visualise the coverage and other mapping statistics (Okonechnikov *et al.* 2016). The consensus genome is generated with samtools (Danecek *et al.* 2021).

### 2.3 Downstream analyses

The user determines whether a phylogenetic analysis should be performed. Phylogenetic trees can be constructed concurrently using both maximum likelihood and Bayesian inference methods. The virus whose sequences are used in the phylogenetic analysis is determined by the largest bit-score after the consensus genomes of all samples have been blasted against the given database. All sequences belonging to the selected virus from the database and the generated consensus genomes of all samples are aligned using MAFFT or Muscle, selected by the user within the config file (Katoh and Standley 2013, Edgar 2022). CIAlign edits the alignment automatically and simultaneously creates plots visualising it before and after editing (Tumescheit *et al.* 2022). The seqmagick tool converts the alignment into NEXUS format afterwards (https://github.com/fhcrc/seqmagick). Since automatic alignment editing may not be sufficient, it is possible to add FASTA files within the input folder. If these are specified together with other samples, they are aligned with the assembled genomes and the viral genomes in the database. If, on the other hand, only FASTA files, e.g. a manually edited alignment, are present in the input folder, the pipeline starts with these sequences alone from the alignment step. To determine the model used in the phylogenetic analysis, the IQ-TREE2 model selection is used, whereby only models that are available in MrBayes are included in the search (Minh *et al.* 2020). IQ-TREE2 uses the selected model to infer the phylogenetic tree by maximum likelihood. The model is automatically translated into the correct notation, so that MrBayes determines a phylogenetic tree using Bayesian inference based on the same type of model (Ronquist *et al.* 2012). The inferred trees are annotated within a NEXUS file which is then visualised with FigTree (https://github.com/rambaut/figtree/). The user also has the option of analysing structural variants within the consensus sequences using Sniffles2 (Smolka *et al.* 2024). VirDetector summarises the most important results visually in a PDF file using fpdf and pypdf (https://github.com/reingart/pyfpdf, https://pypi.org/project/pypdf/).

### 2.4 Metagenomics

Within the metagenomic step, the reads from the individual samples are first mapped against sequences from a database using KMA (Clausen *et al.* 2018). The database used contains viral reference sequences, which we filter during installation using Biopython to limit its size (Cock *et al.* 2009). Depending on the organisms present in the sample, multiple genomes may be assembled. In the subsequent taxonomic classification, Kraken2 assigns a taxonomic label to each assembled genome (Wood *et al.* 2019). The results are summarised in one report and visualised individually for each sample using Krona (Ondov *et al.* 2011).

### 2.5 Configuration

The user determines the steps to be carried out by VirDetector within a config file. If the raw output data of the nanopore sequencing is available and is to be basecalled, the kit and, if applicable, the flow cell type used must be specified in this file. For the execution of the reference-based assembly, the path to the reference sequence must be provided, while the expected genome size must be set for the hybrid assembly approach. It is also possible to change the paths to the respective directories via the config file. For the most important programs, their respective console call can be customised. We provide an example use case in the GitHub repository. The outputs include a summary of the main visual results (Supplementary Material S2) and the assembled viral consensus genomes (Supplementary Material S3).

VirDetector offers a selection of viruses within the given database (Supplementary Table S4). It can however be flexibly extended by simply adding sequences of other viruses or organisms.

## 3 Conclusion

VirDetector is an easily usable yet flexible bioinformatic pipeline for virus surveillance using nanopore sequencing. The installation process is straightforward, with all dependencies automatically installed through Conda or Docker. By integrating all essential steps from basecalling to phylogenetic analysis, the pipeline simplifies the analysis process and ensures accessibility to a wide range of users. VirDetector supports multiple input formats and allows users to customise various steps of the analysis while keeping the number of required parameters to a minimum, balancing flexibility with ease of use. This comprehensive and user-friendly approach makes VirDetector a valuable resource for virus surveillance and research, with applications ranging from routine laboratory data analysis to monitoring outbreaks in the field.

## Supplementary Material

btaf029_Supplementary_Data

## Data Availability

The data underlying this article are publicly available with their sources described in the article.
