# Supplementary material for: VirDetector: a bioinformatic pipeline for virus surveillance using nanopore sequencing
Source: Bioinformatics. 2025 Jan 21;41(2):btaf029. doi: 10.1093/bioinformatics/btaf029 (PMC11802467; doi:10.1093/bioinformatics/btaf029)
Supplement: btaf029_Supplementary_Data [file btaf029_supplementary_data.zip › a8752_supplementary_material.zip!/supplementary_material/Supplementary_Material_2.pdf]

Read quality before trimming

Comparing number of reads

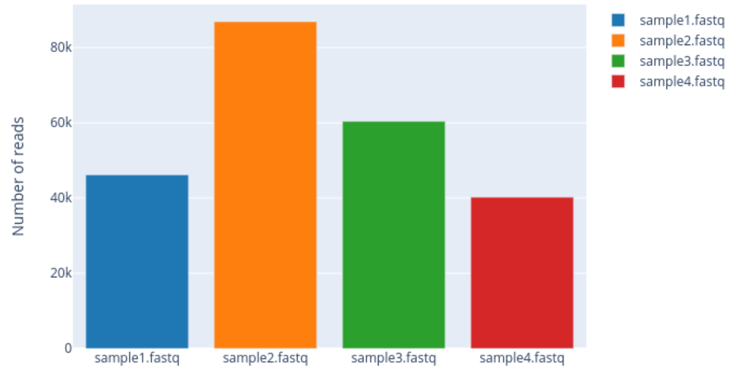

Comparing throughput in bases

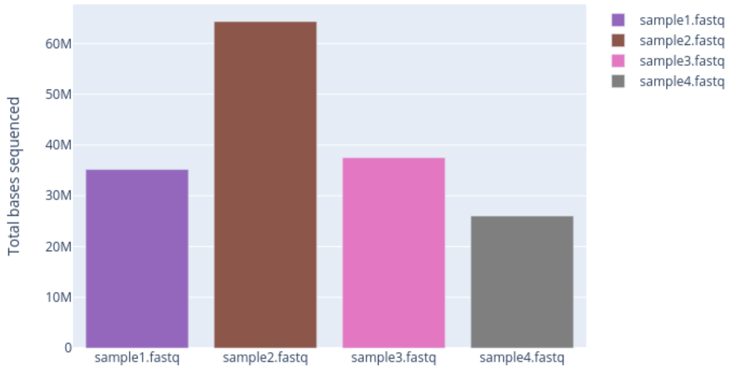

Comparing read length

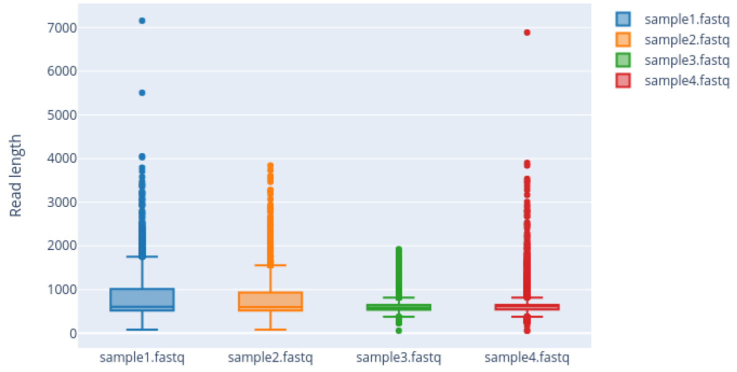

Comparing average base call quality score

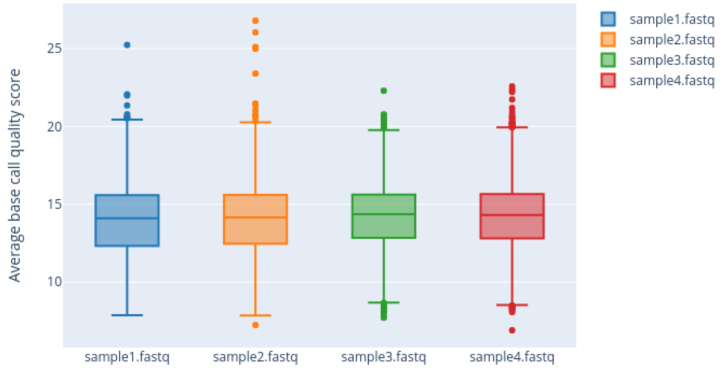

Read quality after trimming

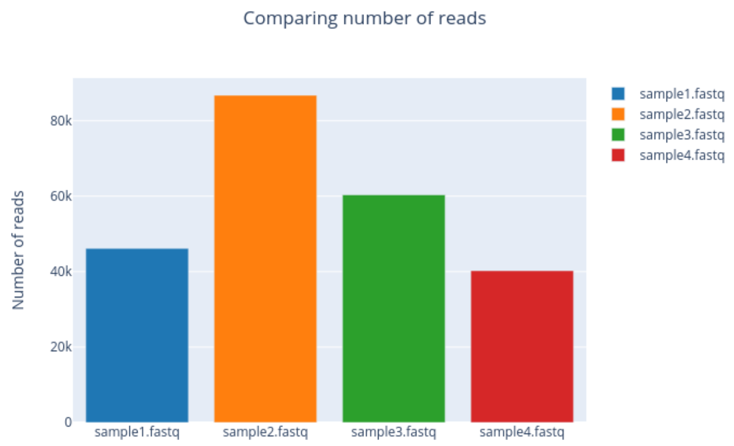

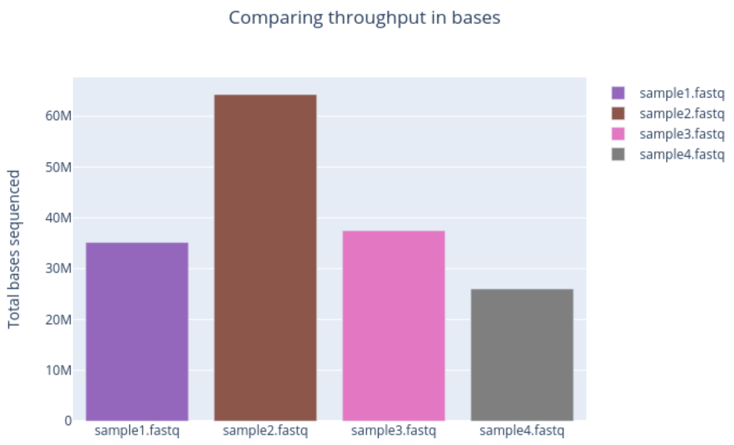

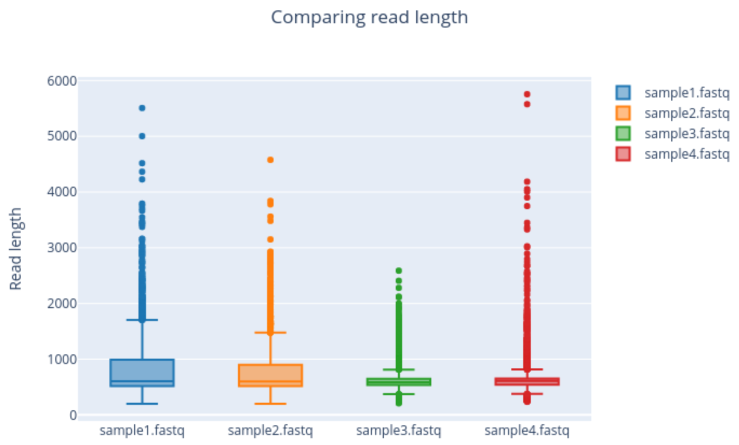

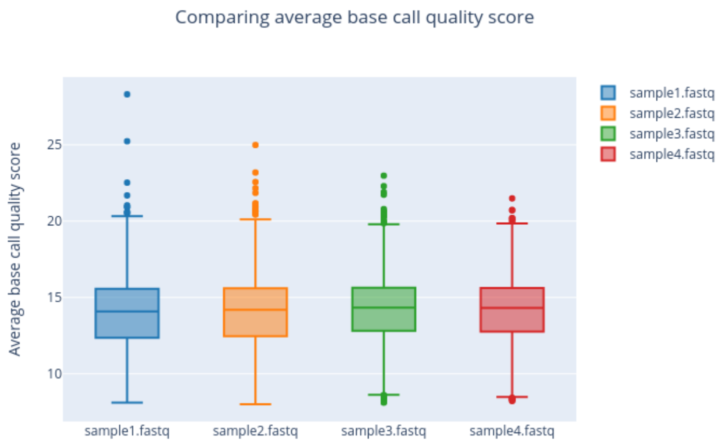

Reference Assembly
Mapping sample3 to MH986055.1

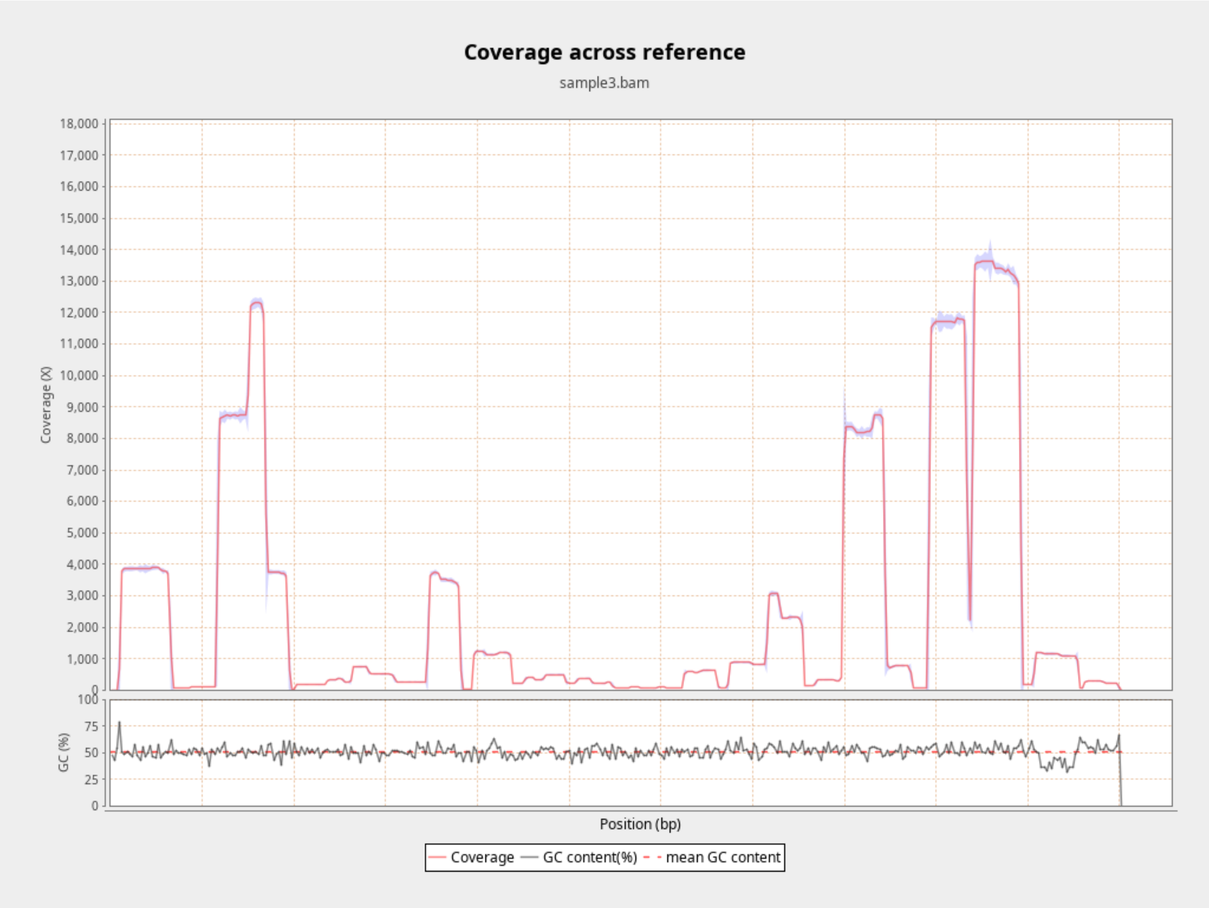

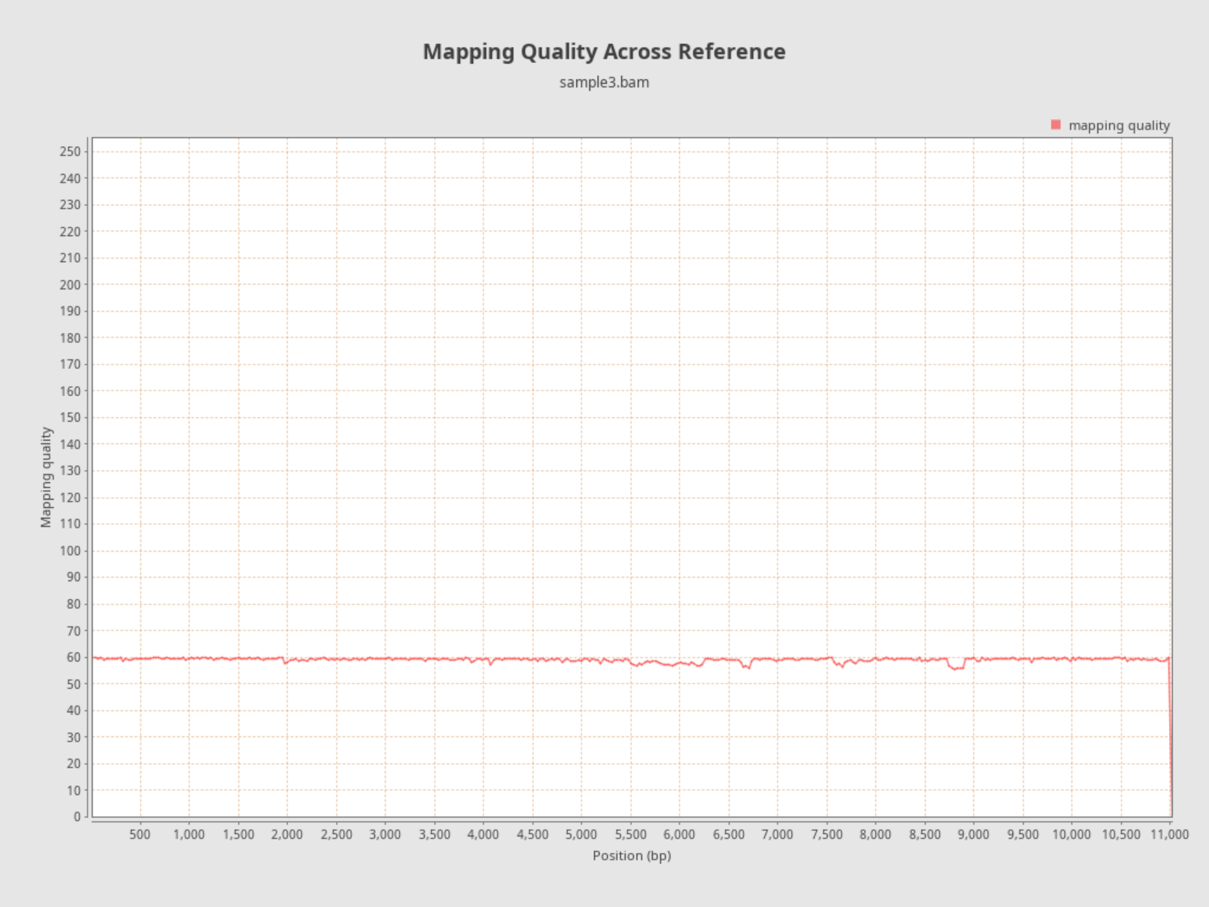

Reference Assembly
Mapping sample1 to MH986055.1

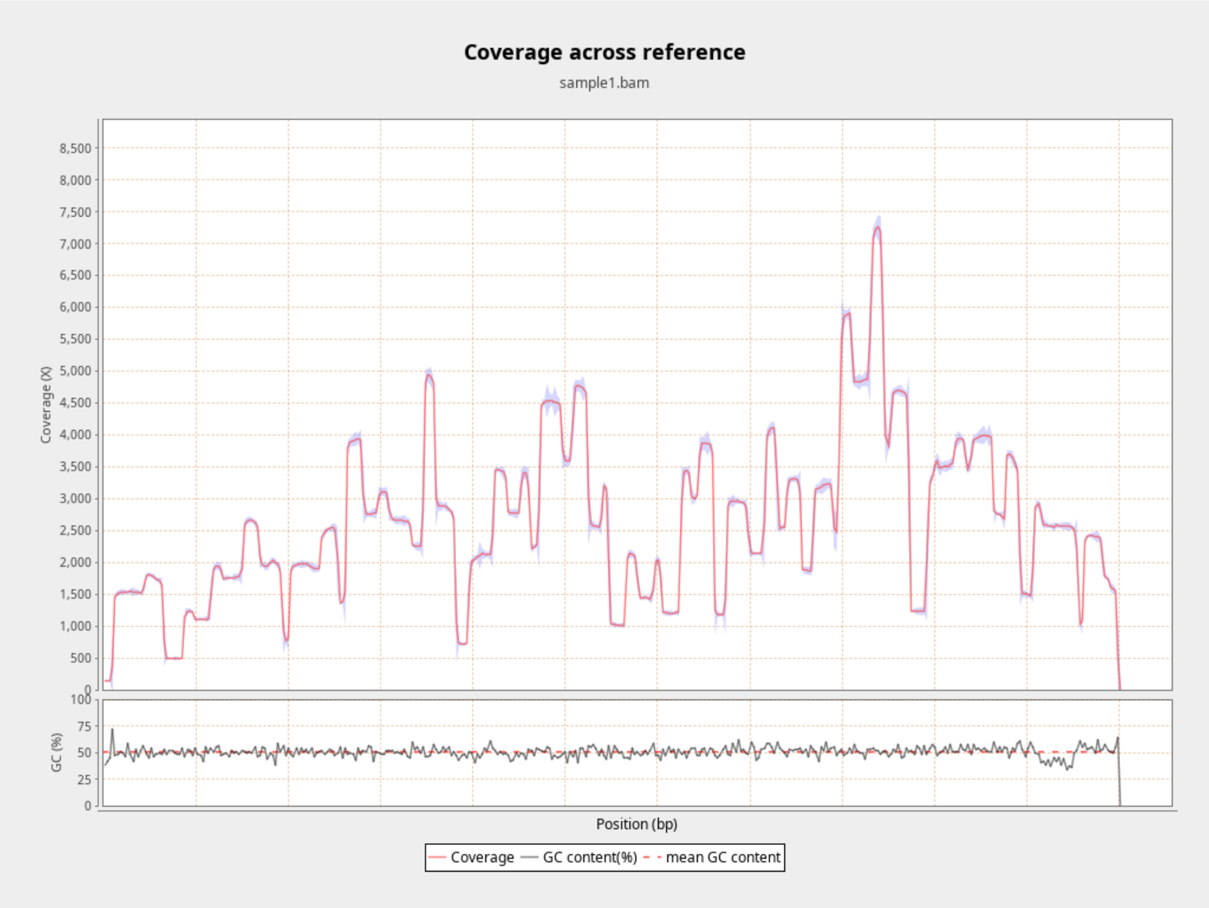

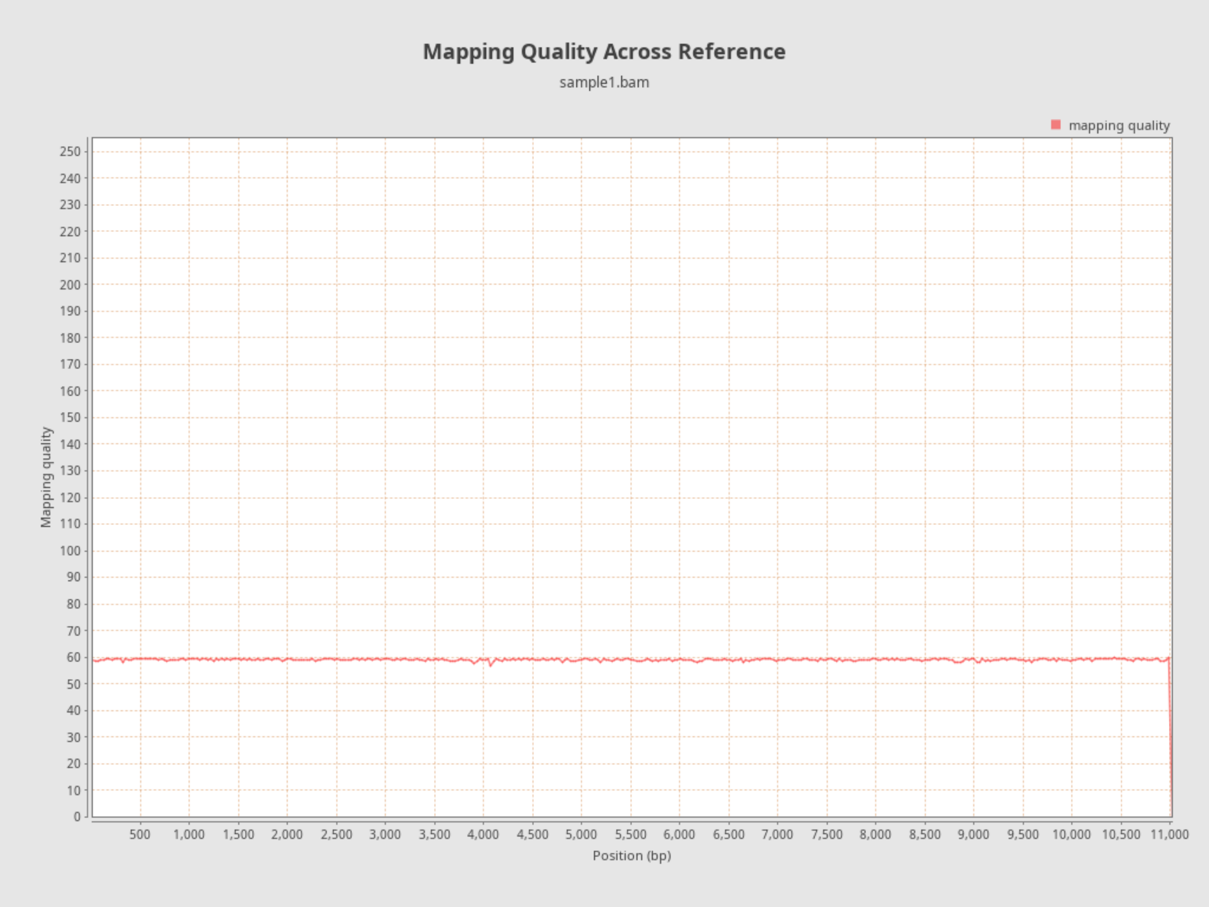

Reference Assembly
Mapping sample4 to MH986055.1

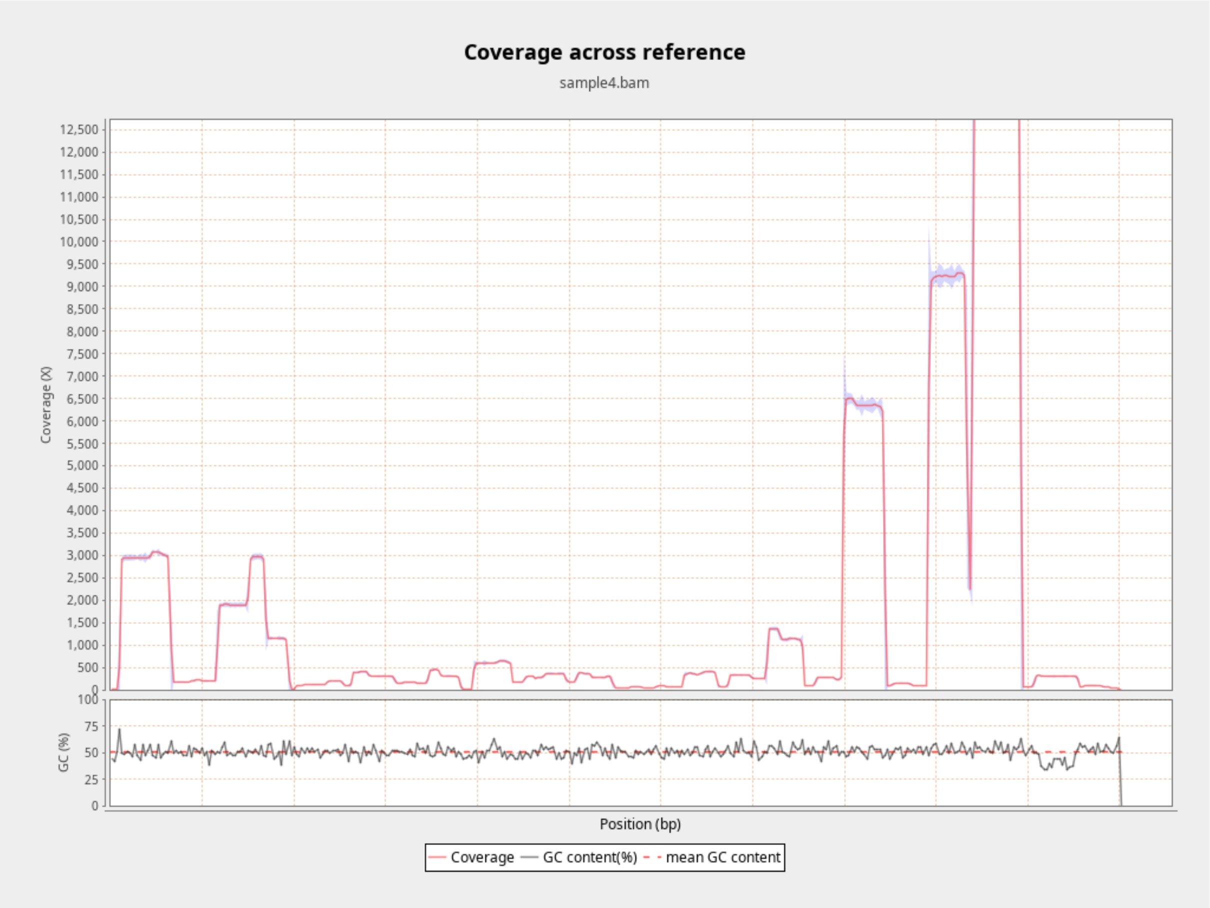

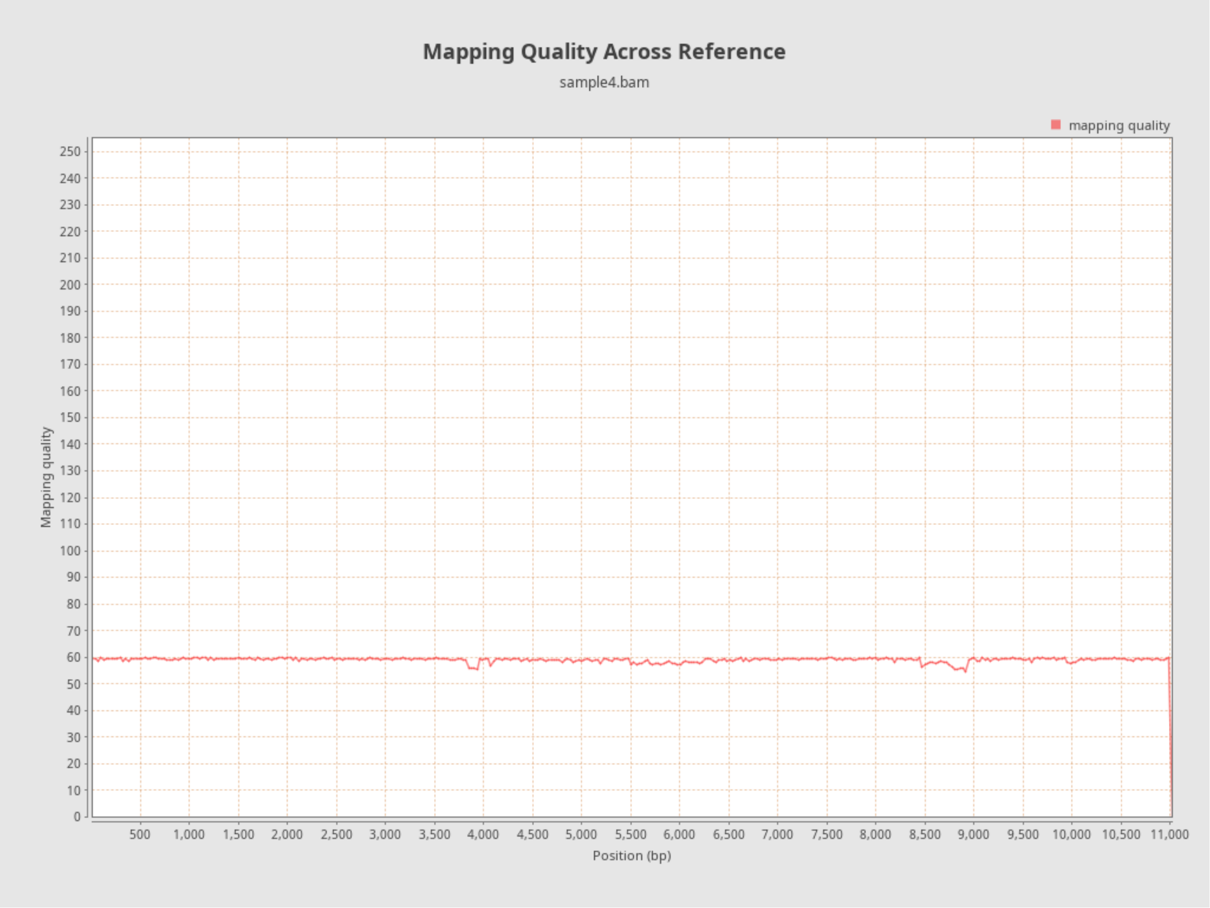

Reference Assembly
Mapping sample2 to MH986055.1

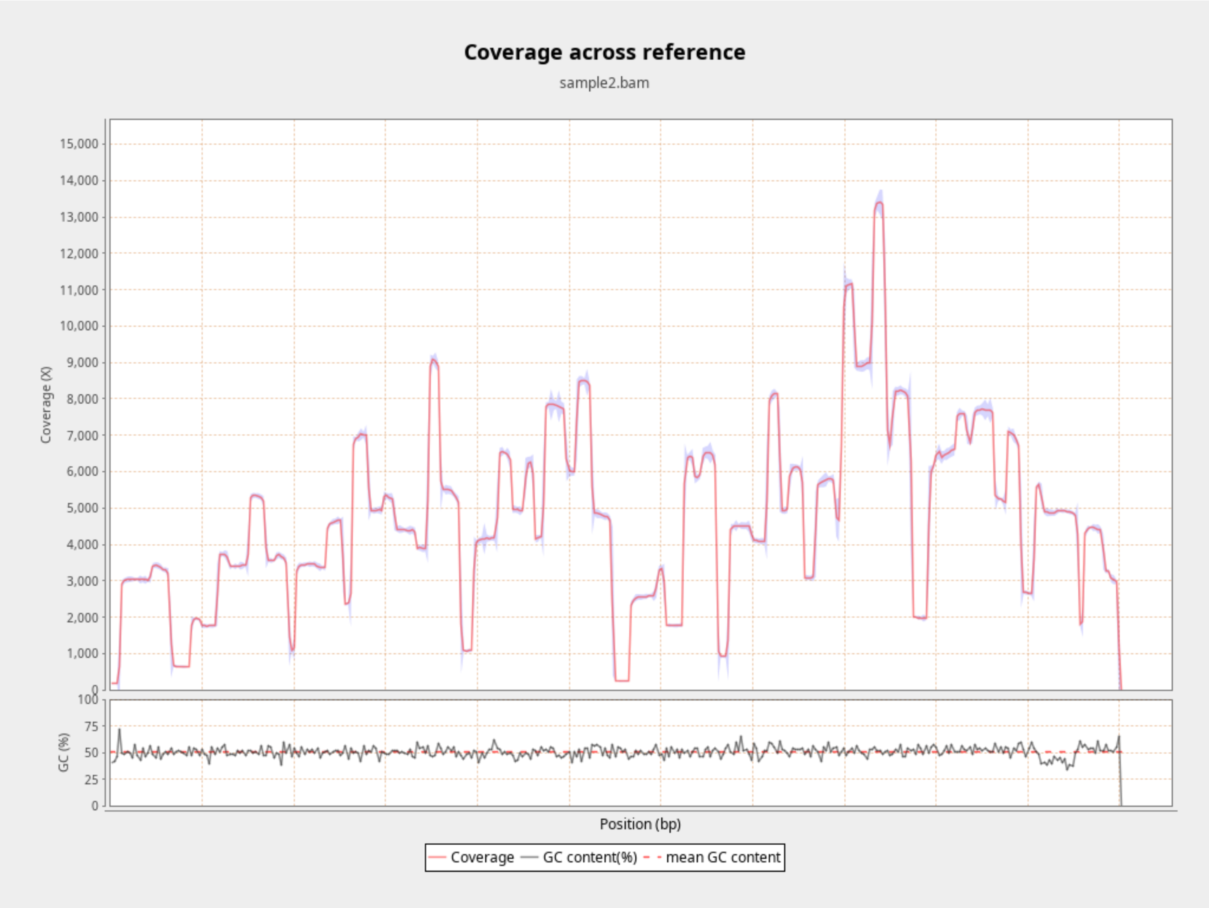

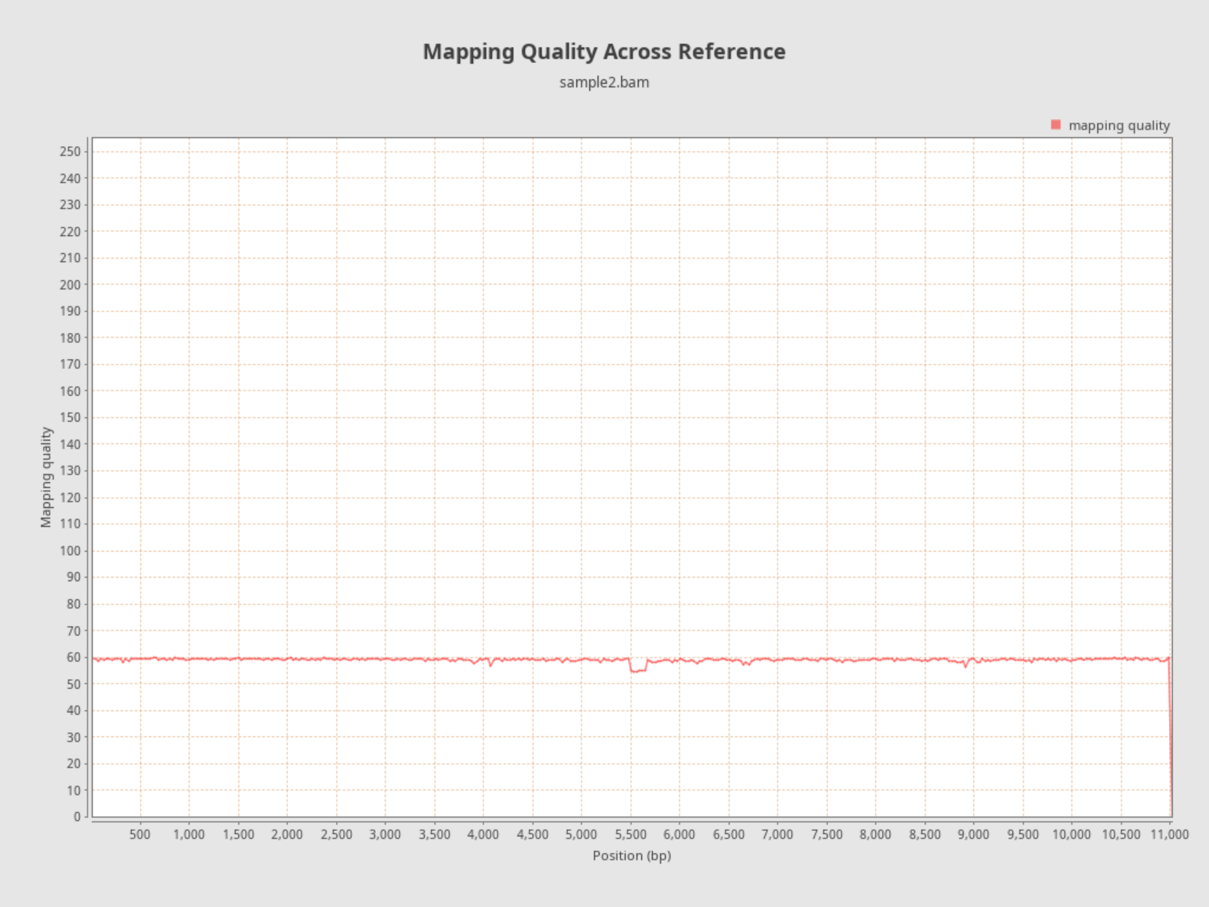

Alignment Overview for Reference Assembly

Before Editing

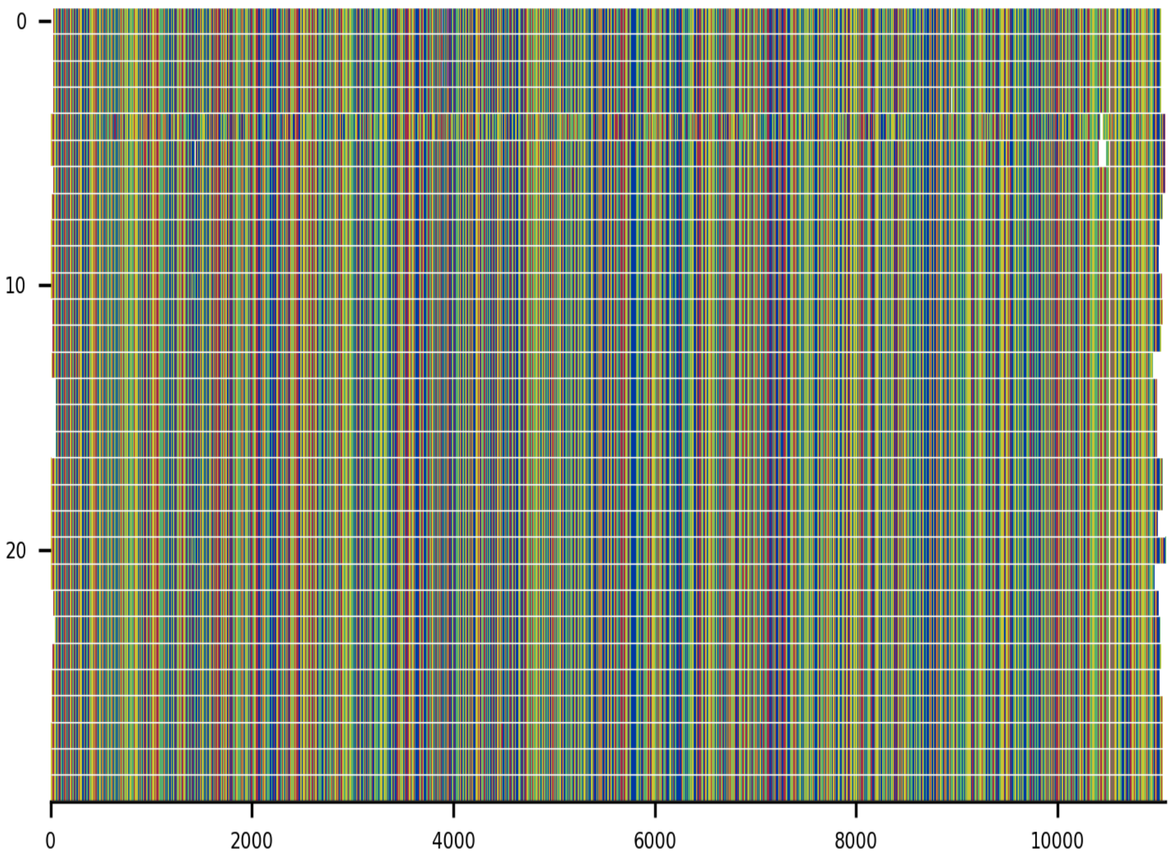

After Editing

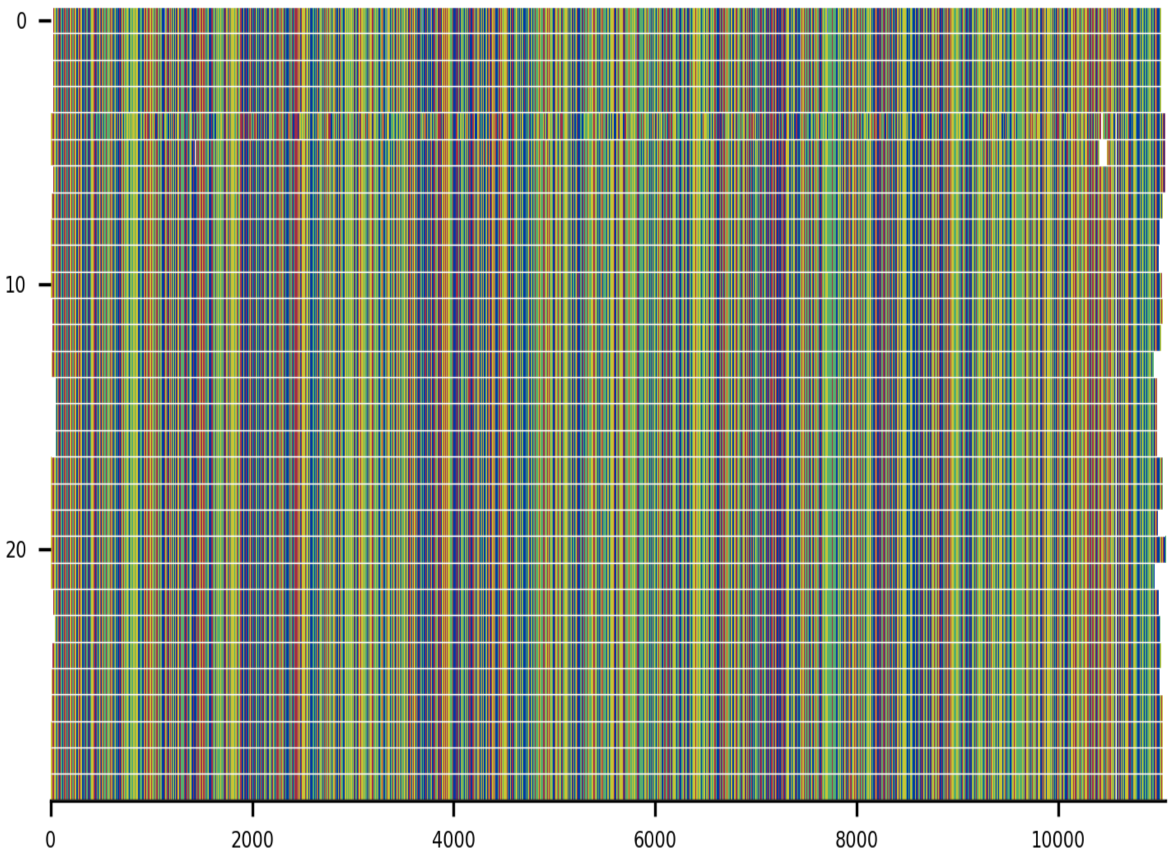

Maximum-Likelihood Tree for Reference Assembly

Values: Bootstrap

Model: GTR+F+R2

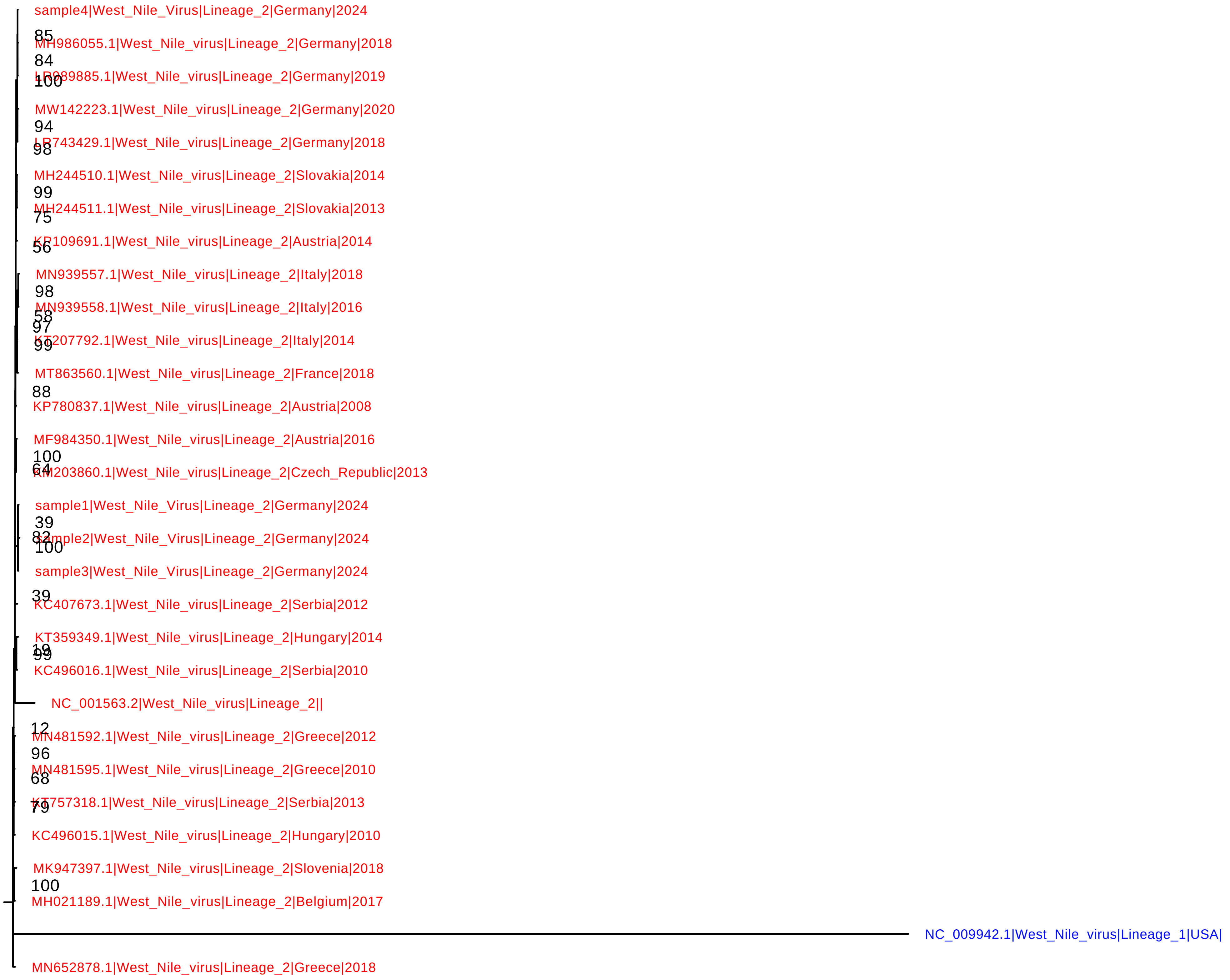
sample4|West_Nile_Virus|Lineage_2|Germany|2024
85
MH986055.1|West_Nile_virus|Lineage_2|Germany|2018
84
100
LR989885.1|West_Nile_virus|Lineage_2|Germany|2019
MW142223.1|West_Nile_virus|Lineage_2|Germany|2020
94
LR743429.1|West_Nile_virus|Lineage_2|Germany|2018
98
MH244510.1|West_Nile_virus|Lineage_2|Slovakia|2014
99
MH244511.1|West_Nile_virus|Lineage_2|Slovakia|2013
75
KP109691.1|West_Nile_virus|Lineage_2|Austria|2014
56
MN939557.1|West_Nile_virus|Lineage_2|Italy|2018
98
MN939558.1|West_Nile_virus|Lineage_2|Italy|2016
58
97
KT207792.1|West_Nile_virus|Lineage_2|Italy|2014
99
MT863560.1|West_Nile_virus|Lineage_2|France|2018
88
KP780837.1|West_Nile_virus|Lineage_2|Austria|2008
MF984350.1|West_Nile_virus|Lineage_2|Austria|2016
100
64
KM203860.1|West_Nile_virus|Lineage_2|Czech_Republic|2013
sample1|West_Nile_Virus|Lineage_2|Germany|2024
39
82
sample2|West_Nile_Virus|Lineage_2|Germany|2024
100
sample3|West_Nile_Virus|Lineage_2|Germany|2024
39
KC407673.1|West_Nile_virus|Lineage_2|Serbia|2012
KT359349.1|West_Nile_virus|Lineage_2|Hungary|2014
19
99
KC496016.1|West_Nile_virus|Lineage_2|Serbia|2010
NC_001563.2|West_Nile_virus|Lineage_2||
12
MN481592.1|West_Nile_virus|Lineage_2|Greece|2012
96
MN481595.1|West_Nile_virus|Lineage_2|Greece|2010
68
KT757318.1|West_Nile_virus|Lineage_2|Serbia|2013
79
KC496015.1|West_Nile_virus|Lineage_2|Hungary|2010
MK947397.1|West_Nile_virus|Lineage_2|Slovenia|2018
100
MH021189.1|West_Nile_virus|Lineage_2|Belgium|2017
NC_009942.1|West_Nile_virus|Lineage_1|USA|
MN652878.1|West_Nile_virus|Lineage_2|Greece|2018
