# Supplementary material for: VirDetector: a bioinformatic pipeline for virus surveillance using nanopore sequencing
Source: Bioinformatics. 2025 Jan 21;41(2):btaf029. doi: 10.1093/bioinformatics/btaf029 (PMC11802467; doi:10.1093/bioinformatics/btaf029)
Supplement: btaf029_Supplementary_Data [file btaf029_supplementary_data.zip › a8752_supplementary_material.zip!/supplementary_material/Supplementary_Material_3.docx]

Consensus genome Sample 1:

>sample1
ACAAACTTAGTAGTGTTTGTGAGGATTAATAACAATTAACACAGTGCGAGCTGTTTCTTAGCACGAAGATCTCGATGTCTAAGAAACCAGGAGGGCCCGGTAAAAACCGGGCTGTCAATATGCTAAAACGCGGTATGCCCCGCGGATTGTCCTTGATAGGACTGAAGAGGGCTATGCTGAGTCTGATTGACGGGAAAGGCCCAATACGTTTCGTGTTGGCTCTTTTGGCGTTTTTCAGATTCACTGCAATCGCTCCGACTCGTGCGGTGCTGGACAGATGGAGAGGCGTCAACAAACAAACAGCGATGAAGCATCTCTTGAGTTTCAAGAAAGAACTAGGAACTCTGACCAGTGCCATCAACCGCCGGAGCACAAAACAAAAGAAAAGAGGAGGCACAGCGGGCTTTACTATCTTGCTTGGGCTGATCGCTTGTGCTGGAGCTGTGACCCTCTCNAACTTCCAGGGCAAAGTGATGATGACAGTCAATGCAACCGATGTTACTGACGTGATTACCATCCCAACAGCTGCTGGGAAAAACCTGTGCATTGTGAGGGCTATGGACGTGGGATACCTTTGTGAGGATACTATCACTTATGAATGTCCGGTCCTAGCTGCTGGAAATGACCCTGAAGACATTGACTGCTGGTGCACGAAATCATCTGTTTACGTGCGCTATGGAAGATGCACAAAAACTCGCCATTCCCGTCGAAGCAGAAGGTCTCTGACTGTCCAGACACATGGAGAAAGCACACTGGCCAACAAGAAAGGAGCTTGGTTGGACAGTACAAAAGCCACGAGGTATCTGGTGAAGACAGAATCATGGATACTGAGAAACCCGGGCTACGCCCTCGTTGCAGCGATCATTGGATGGATGCTAGGAAGCAACACAATGCAACGCGTCGTGTTTGCCATCCTATTGCTCCTGGTGGCACCAGCATACAGCTTCAATTGCTTGGGAATGAGTAACAGAGACTTCCTGGAGGGAGTGTCTGGAGCTACATGGGTTGATCTGGTACTGGAAGGCGATAGTTGTGTGACCATAATGTCAAAGGACAAGCCAACCATTGATGTTAAAATGATGAGCATGGAAGCAACCAACCTCGCGGATGTGCGCAGCTACTGTTACCTAGCTTCGGTCAGTGACTTGTCAACGAGAGCCGCGTGTCCAACCATGGGTGAAGCCCACAACGAGAAAAGAGCTGACCCCGCCTTCGTTTGCAAGCAAGGCGTTGTGGACAGAGGATGGGGAAATGGCTGCGGACTGTTTGGAAAGGGAAGCATTGACACATGCGCGAAGTTTGCCTGTACAACCAAAGCAACTGGATGGATCATCCAGAAGGAGAACATCAAGTATGAGGTCGCCATATTTGTGCATGGCCCGACGACTGTTGAATCTCATGGCAATTATTCAACACAGATGGGGGCCACCCAGGCTGGAAGATTCAGTATAACTCCATCGGCGCCATCTTACACGCTAAAGTTGGGTGAGTATGGTGAGGTCACGGTTGACTGTGAGCCACGGTCAGGAATAGACACTAGCGCCTATTACGTTATGTCAGTTGGTGCGAAGTCCTTCCTGGTTCACCGAGAATGGTTTATGGATCTGAACCTGCCATGGAGCAGTGCTGGAAGCACCACGTGGAGGAATCGGGAAACACTGATGGAGTTTGAAGAACCTCATGCCACCAAACAATCTGTTGTGGCTCTAGGGTCGCAGGAAGGTGCATTGCACCAAGCTCTGGCCGGAGCGATTCCTGTTGAGTTCTCAAGCAATACTGTGAAGTTGACATCAGGACATCTGAAGTGCAGGGTGAAGATGGAGAAGTTGCAGCTTAAGGGAACAACATATGGAGTATGTTCAAAAGCGTTTAAATTCGCTGGGACTCCCGCTGACACTGGCCATGGAACGGTGGTGTTGGAACTGCAATACACCGGAACAGACGGTCCCTGCAAAGTGCCCATTTCTTCCGTAGCTTCTCTGAATGACCTCACACCTGTTGGAAGACTGGTGACCGTGAATCCATTTGTGTCTGTGGCCACAGCCAACTCGAAGGTCTTGATTGAACTCGAACCCCCGTTTGGTGACTCTTACATCGTGGTGGGAAGAGGAGAACAGCAGATAAACCACCACTGGCACAAATCTGGGAGCAGCATCGGAAAGGCCTTTACCACTACACTCAGAGGAGCTCAACGACTCGCAGCTCTTGGAGACACTGCTTGGGATTTTGGGTCAGTTGGAGGGGTTTTCACCTCAGTGGGGAAAGCCATACACCAAGTCTTTGGAGGAGCTTTTAGATCACTCTTTGGAGGGATGTCCTGGATCACACAGGGACTTTTAGGAGCCCTTCTGTTGTGGATGGGAATCAATGCNCGTGACAGGTCAATTGCCATGACGTTTCTTGCGGTTGGAGGAGTTTTGCTCTTCCTTTCGGTCAACGTCCATGCTGACACAGGCTGTGCCATTGATATTGGCAGGCAAGAGCTCCGGTGTGGAAGTGGAGTGTTTATCCACAACGATGTGGAAGCCTGGATGGATCGTTACAAGTTCTACCCGGAGACACCACAAGGCCTAGCAAGAATCATTCAGAAAGCACATGCAGAAGGAGTCTGCGGCTTGCGTTCCGTTTCCAGACTCGAGCACCAAATGTGGGAAGCTATTAAGGATGAGCTGAACACCCTGTTGAAAGAGAATGGAGTCGACTTGAGTGTCGTGGTGGAAAANCAAAATGGGATGTATAAAGCAGCACCAAAACGTTTGGCTGCTACCACCGAAAAACTGGAGATGGGTTGGAAGGCTTGGGGCAAGAGCATCATCTTTGCTCCAGAACTAGCTAACAACACCTTTGTCATCGACGGTCCTGAGACTGAGGAATGCCCAACGGCCAACCGAGCATGGAACAGTATGGAGGTAGAAGACTTTGGATTTGGATTGACAAGCACTCGCATGTTCCTGAGGATTCGGGAAACGAACACAACNGAATGCGACTCGAAGATCATAGGAACCGCCGTCAAGAACAACATGGCTGTGCATAGTGATCTGTCGTACTGGATAGAGAGCGGACTCAACGACACCTGGAAGCTTGAGAGGGCGGTTCTAGGAGAAGTCAAATCATGCACCTGGCCAGAAACCCACACTTTGTGGGGTGATGGAGTTCTGGAAAGTGATCTCATCATACCCATCACCTTGGCAGGACCCAGAAGCAATCATAACAGGAGACCAGGGTACAAAACTCAGAACCAAGGTCCATGGGATGAGGGGCGTGTCGAGATTGACTTTGACTATTGCCCAGGAACAACAGTAACTATAAGTGANAGTTGCGGACACCGTGGACCTGCGGCACGCACAACCACTGAGAGTGGGAAGCTCATTACAGACTGGTGCTGCAGAAGCTGCACCCTCCCTCCACTGCGCTTCCAGACTGAAAATGGCTGTTGGTACGGAATGGAAATTCGACCCACGCGGCACGACGAAAAGACCCTCGTGCAATCGAGAGTGAATGCATACAACGCCGACATGATTGATCCTTTTCAGTTGGGCCTTCTGGTCGTGTTCTTGGCCACCCAGGAGGTCCTTCGCAAGAGGTGGACGGCCAAGATCAGCATTCCAGCTATCATGCTTGCACTCCTAGTCCTAGTGTTTGGGGGTATTACGTACACTGATGTCCTGCGATATGTCATTCTCGTCGGCGCCGCGTTTGCTGAAGCAAACTCAGGAGGAGACGTTGTGCACTTGGCACTCATGGCCACATTCAAGATTCAACCAGTCTTTCTGGTGGCTTCCTTTTTGAAGGCAAGGTGGACCAACCAAGAGAGTATTTTGCTCATGCTTGCAGCTGCTTTCTTTCAAATGGCTTACTATGATGCCAAGAATGTTTTGTCATGGGAAGTGCCTGACGTTTTGAACTCTCTCTCCGTCGCATGGATGATTCTCAGAGCTATAAGCTTCACCAACACNTCAAATGTGGTGGTGCCGCTGCTGGCCCTTCTGACACCTGGACTAAAATGCCTAAACCTTGATGTGTACAGAATTTTGCTACTCATGGTTGGAGTTGGAAGCCTCATCAAAGAAAAAGGAGCTCTGCAGCAAAAAAGAAAGGGGCTTGCCTCATCTGCCTAGCGCTGGCGTCTACAGGAGCGTTCAATCCAATGATACTTGCAGCTGGGCTGATGGCTTGCGACCCCAACCGCAAGCGGGGCTGGCCTGCCACAGAAGTGATGACTGCAGTTGGACTCATGTTTGCCATCGTTGGGGGTCTGGCAGAACTTGACATAGACTCTATGGCTATCCCCATGACCATTGCCGGACTCATGTTCGTGGCATTTGTCATCTCTGGAAAGTCAACAGACATGTGGATTGAGAGGACGGCTGATATTACTTGGGAGAGTGACGCTGAAATCACAGGCTCTAGTGAAAGAGTAGATGTTAGGCTGGATGATGATGGAAATTTTCAATTGATAAATGACCCTGGGGCGCCATGGAAAATTTGGATGCTTAGGATGGCCTGCCTGGCAATAAGTGCCTACACACCTTGGGCGATTCTTCCCTCGGTCATTGGATTCTGGATAACCCTTCAGTACACAAAGAGAGGAGGTGTCCTTTGGGACACACCATCACCCAGGGAGTACAAGAAGGGTGATACCACCACTGGCGTTTACAGAATCATGACTCGAGGTTTGCTTGGCAGTTACCAAGCTGGAGCCGGAGTGATGGTAGAGGGAGTGTTCCACACACTATGGCACACCACTAAGGGAGCTGCTCTCATGAGTGGTGAAGGACGTCTGGATCCCTATTGGGGGAGCGTGAAAGAAGACCGGCTTTGCTATGGGGGGCCATGGAAACTCCAACATAAATGGAATGGACATGATGAGGTCCAAATGATTGTTGTGGAGCCAGGAAAAAATGTGAAAAACGTCCAGACTAAGCCCGGAGTGTTTAAGACACCAGAAGGAGAAATTGGGGCAGTTACGCTAGACTATCCCACTGGAACGTCAGGTTCCCCCATTGTGGATAAAAATGGAGATGTGATTGGATTGTATGGGAACGGCGTCATCATGCCTAATGGTTCATACATAAGCGCCATTGTGCAAGGAGAGAGAATGGAAGAACCGGCACCAGCTGGCTTCGAACCTGAAATGTTGAGGAAGAAACAGATCACTGTCCTTGATCTGCACCCCGGAGCAGGAAAGACGCGCAAGATACTTCCCCAAATCATCAAGGAGGCCATCAACAAAAGANTGAGGACGGCTGTACTGGCACCCACCAGGGTCGTTGCTGCTGAGATGTCTGAGGCCCTGAGAGGACTTCCCATCCGGTACCAAACCTCAGCAGTGCACAGAGAGCACAGTGGAAATGAGATCGTTGATGTCATGTGCCATGCCACTCTCACACACAGGCTGATGTCTCCACACAGAGTCCCCAATTACAATCTGTTCATAATGGATGAAGCCCATTTCACGGATCCAGCGAGCATCGCAGCCAGAGGATACATAGCAACCAAGGTTGAATTGGGCGAAGCCGCCGCGATTTTCATGACGGCAACGCCACCCGGGACTTCTGACCCCTTTCCAGAGTCCAATGCTCCTATCTCGGACATGCAAACAGAGATCCCAGACAGAGCCTGGAACACTGGATATGAATGGATAACTGAGTATGTTGGAAAGACCGTTTGGTTTGTTCCAAGTGTGAAAATGGGAAATGAAATTGCCCTCTGTCTGCAACGGGCGGGGAAGAAGGTTATCCAGCTGAACAGAAAGTCCTATGAGACAGAGTACCCCANGTGCAAGAACGATGATTGGGATTTTGTCATCACCACAGACATATCAGAAATGGGAGCAAACTTCAAGGCAAGCAGAGTGATCGACAGCCGCAAAAGCGTGAAACCCACCATCATTGAGGAAGGTGATGGAAGAGTCATTCTGGGGGAACCCTCAGCCATCACGGCTGCCAGCGCTGCTCAGCGGAGAGGACGCATAGGAAGAAACCCATCACAAGTTGGTGATGAGTATTGCTATGGAGGGCACACAAATGAGGATGATTCCAACTTTGCCCACTGGACAGAGGCTCGCATCATGCTAGACAACATCAACATGCCGAATGGTCTGGTGGCCCAACTATATCAGCCTGAGCGCGAGAAGGTGTACACCATGGACGGGGAATATAGGCTCAGAGGGGAAGAACGGAAGAACTTCCTTGAATTCCTGAGAACAGCTGATTTACCAGTCTGGCTCGCTTACAAAGTGGCAGCAGCAGGAATATCATACCATGACCGGAAGTGGTGCTTTGATGGACCTCGAACCAACACGATTCTTGAAGACAACAATGAAGTTGAAGTCATCACGAAGTTGGGTGAGAGAAAGATCTTAAGACCCAGGTGGGCGGATGCCAGAGTGTACTCAGACCACCAAGCTCTAAAGTCCTTCAAAGATTTTGCATCAGGGAAACGATCACAAATCGGGCTCGTTGAGGTGCTCGGGAGAATGCCTGAGCACTTCATGGGGAAAACTTGGGAGGCGTTGGACACGATGTATGTGGTGGCAACCGCTGAAAAAGGAGGCCGAGCTCACAGGATGGCTCTTGAGGAGCTACCGGACGCCCTTCAGACAATAGCTTTGATTGCACTATTGAGTGTGATGTCCTTAGGTGTGTTTTTTCTACTCATGCAAAGGAAAGGCATTGGTAAGATTGGCTTGGGAGGAGTGATCTTAGGAGCTGCCACATTCTTCTGCTGGATGGCTGAAGTCCCAGGAACGAAAATAGCAGGCATGCTCCTGCTTTCCCTGCTGCTCATGATCGTTTTGATTCCGGAGCCGGAAAAGCAGCGCTCACAGACTGACAACCAGCTTGCCGTGTTCTTGATTTGTGTGCTCACACTGGTCAGCGCCGTGGCTGCCAATGAAATGGGTTGGCTGGACAAGACCAAGAATGACATCAGCAGCCTGTTGGGACACAAGCCAGAAACTAGAGAGACGACCCTGGGAGTTGAGAACTTCTTGCTTGATCTGCGGCCTGCTACAGCATGGTCGCTCTATGCCGTAACGACAGCCGTCCTTACCCCTTTGCTGAAACATCTAATTACGTCAGATTACATCAACACTTCGCTGACCTCAATAAACGTCCAAGCCAGTGCGTTGTTCACCTTGGCCAGAGGCTTCCCTTTTGTGGACGTTGGTGTGTCAGCTCTCTTGCTGGCGGCCGGGTGCTGGGGCCAAGTGACCCTGACTGTGACTGTGACTGCAGCTGCCCTGCTCTTTTGCCACTATGCTTACATGGTACCAGGCTGGCAAGCGGAAGCCATGCGATCCGCCCAGCGGCGGACAGCTGCTGGCATCATGAAAAATGCAGTGGTGGATGGGATCGTGGCTACCGATGTACCTGAACTTGAGCGAACAACTCCAGTCATGCAGAAAAANGTTGGACAGATCATGCTGATCTTGGTGTCAATGGCCGCGGTTGTCGTCAATCCATCAGTGAGAACTGTCAGAGAGGCTGGAATTCTGACTACAGCAGCAGCAGTCACCTTATGGGAGAATGGTGCTAGTTCAGTGTGGAATGCAACGACAGCTATTGGGCTTTGTCACATCATGCGAGGAGGATGGCTCTCGTGTCTCTCCATCACGTGGACTCTCATCAAAAACATGGAAAAACCAGGCCTCAAGAGGGGTGGAGCCAAGGGACGCACGCTAGGGGAAGTTTGGAAGGAGAGACTCAACCACATGACGAAGGAAGAATTTACCAGATACAGAAAAGAAGCCATCACTGAAGTCGACNGCTCCGCGGCAAAACATGCCAGGAGAGAGGGAAACATCACTGGAGGCCATCCAGTCTCACGGGGAACCGCGAAATTACGGTGGTTGGTGGAAAGGCGTTTCCTCGAGCCAGTGGGAAAGGTTGTGGATCTCGGATGTGGTAGAGGCGGCTGGTGTTATTACATGGCTACCCAGAAGAGGGTACAGGAAGTGAAAGGGTACACGAAAGGAGGACCTGGCCATGAAGAGCCACAACTGGTGCAGAGCTATGGTTGGAATATTGTTACCATGAAGAGCGGAGTCGACGTCTTCTACAGACCATCAGAAGCGAGCGACACACTGCTCTGTGACATTGGAGAGTCATCGTCAAGTGCCGAGGTAGAAGAACACCGCACCGTCCGTGTCTTGGAGATGGTGGAAGACTGGCTGCACAGAGGACCTAAGGAATTCTGCATCAAAGTGCTATGCCCTTACATGCCCAGAGTGATTGAGAAGATGGAAACACTCCAAAGGCGATATGGAGGTGGCCTTGTGAGAAACCCCCTTTCACGCAACTCTACCCATGAGATGTACTGGGTGAGCCACGCTTCAGGCAACATTGTCCACTCCGTGAACATGACAAGCCAGGTGCTTCTGGGGAGGATGGAAAAGAAAACATGGAAGGGACCCCAGTTTGAGGAAGATGTNAACTTGGGAAGTGGAACNNGGGCAGTAGGGAAGCCTCTCCTCAATTCTGATACTAGCAAGATCAAGAGCCGAATTGAGAGGCTGAAGAAAGAATACAGCTCCACATGGCACCAGGATGCGAATCACCCCTACAGGACCTGGAACTACCACGGAAGCTATGAAGTGAAACCAACCGGCTCAGCCAGCTCCCTTGTGAATGGGGTAGTTAGATTACTCTCAAAACCATGGGACACTATCACCAATGTGACCACGATGGCCATGACAGACACCACTCCTTTCGGTCAACAACGAGTGTTCAAGGAAAAGGTGGATACAAAGGCTCCAGAGCCTCCAGAAGGGGTCAAATACGTCCTCAATGAGACCACGAACTGGCTGTGGGCTTTTCTAGCCCGCGATAAGAAACCCAGGATGTGTTCCCGGGAGGAATTCATTGGAAAAGTCAACAGTAATGCCGCCCTGGGAGCGATGTTTGAAGAACAGAACCAATGGAAGAACGCCCGGGAAGCCGTGGAGGATCCAAAGTTTTGGGAGATGGTGGATGAGGAGCGTGAGGCGCATCTCCGTGGAGAATGCAACACCTGCATCTACAACATGATGGGAAAGAGAGAGAAGAAGCCTGGAGAGTTCGGCAAAGCTAAAGGCAGCAGAGCCATTTGGTTCATGTGGCTAGGGGCCCGCTTCCTGGAGTTTGAAGCTCTCGGATTCCTCAATGAAGACCACTGGCTGGGTAGGAAGAACTCAGGAGGAGGAGTTGAAGGCTTAGGACTGCAGAAGCTTGGGTACATCTTGAAGGAAGTCGGAACAAAGCCTGGAGGAAAGATCTATGCCGATGATACCGCAGGCTGGGACACACGCATCACCAAAGCTGACCTCGAGAACGAAGCGAAGGTTCTTGAACTGCTGGACGGAGAACACCGACGCCTAGCGCGGTCCATCATCGAGCTCACATACCGACACAAAGTCGTGAAAGTGATGAGGCCAGCGGCCGACGGGAAAACTGTGATGGATGTCATCTCCAGAGAGGATCAGAGAGGAAGCGGGCAGGTAGTGACTTACGCCCTGAACACCTTCACTAATCTAGCAGTTCAGCTGGTCAGAATGATGGAGGGGGAGGGGGTCATTGGACCTGACGATGTTGAAAAACTGGGAAAAGGAAAAGGCCCTAAGGTCAGAACCTGGCTGTTTGAGAATGGNGAGGAGCGTCTCAGTCGCATGGCCGTCAGCGGTGATGACTGCGTGGTGAAACCTTTGGACGACCGCTTCGCCACATCACTACACTTCCTAAATGCCATGTCAAAGGTCCGCAAGGACATCCAGGAATGGAAACCCTCGACAGGGTGGTATGACTGGCAGCAGGTCCCATTCTGTTCAAACCATTTCACGGAACTGATCATGAAGGATGGCAGGACGTTGGTGGTCCCGTGTCGTGGACAAGACGAGTTGATTGGACGGGCCAGAATCTCTCCAGGGGCTGGATGGAATGTGCGCGACACCGCCTGCCTGGCGAAGTCATACGCGCAGATGTGGCTGTTGCTTTATTTTCACCGTAGAGACCTGAGATTGATGGCTAACGCCATCTGTTCCGCTGTGCCTGTCAACTGGGTTCCCACAGGGCGTACCACCTGGTCGATCCACGCAAAAGGAGAATGGATGACGACAGAAGACATGCTCGCAGTCTGGAACAGAGTGTGGATTGAGGAGAATGAGTGGATGGAAGACAAAACACCAGTTGAGAGGTGGAGTGATGTTCCATACTCTGGAAAGAGAGAAGACATTTGGTGTGGCAGTTTGATTGGCACACGAACCCGCGCTACTTGGGCTGAAAATATCCATGTGGCAATCAATCAGGTCCGTTCGGTGATTGGAGAAGAGAAGTATGTGGATTACATGAGCTCTCTGAGGAGGTATGAAGACACCATTGTAGTTGAGGACACTGTTTTGTAAATAATAAAGCTGTATTGAGTAGTTGTACAGTTGTAGTGTTTATAGCAATTTGAATTATGATTAATTATTTAGGCTTAAGATAGTATTATAGTTAGTTTAGTGTAAATAGGTTTTATTGAGAATGGAAGTCAGGCCAGATTAATGCTGCCACCGGAAGTTGAGTAGACGAGGTGCTGCCTGCGGCTCAACCCCAGGAGGACTGGGTGACCAAAGCTGCGAGGTGATCCACGTAAGCCCTCAGAACCGTCTCGGAAGGAGGACCCCACGTGCTTTAGCCTCAAAGCCCAGTGTCAGACCACACTCTAGTGTGCCACTCTGCGGAGAGTGCAGTCTGCGATAGTGCCCCAGGTGGACTGGGTTAACAAAGGCAAAACATCGCCCCACGCGGCCATAACCCTGGCTATGGTGTTAACCAGGGAGAAGGGACTAGAGGTTAGAGGAGACCCCGCGTCAAAAAGTGCACGGCCCAACTTGGCTGAAGCTGTAAGCCAAGGGAAGGACTAGAGGTTAGAGGAGACCCCGTGCCAAAAACACCAAAAGAAACAGCATATTGACACCTGGGATAGACTAGGGGATCTTCTGCTCTGCACAACCAGCCACACGGCACAGTGCGCC

Consensus genome Sample 2:

>sample2
ACAAACTTAGTAGTGTTTGTGAGGATTAATAACAATTAACACAGTGCGAGCTGTTTCTTAGCACGAAGATCTCGATGTCTAAGAAACCAGGAGGGCCCGGTAAAAACCGGGCTGTCAATATGCTAAAACGCGGTATGCCCCGCGGATTGTCCTTGATAGGACTGAAGAGGGCTATGCTGAGTCTGATTGACGGGAAAGGCCCAATACGTTTCGTGTTGGCTCTTTTGGCGTTTTTCAGATTCACTGCAATCGCTCCGACTCGTGCGGTGCTGGACAGATGGAGAGGCGTCAACAAACAAACAGCGATGAAGCATCTCTTGAGTTTCAAGAAAGAACTAGGAACTCTGACCAGTGCCATCAACCGCCGGAGCACAAAACAAAAGAAAAGAGGAGGCACAGCGAGCTTTACTATCTTGCTTGGGCTGATCGCTTGTGCTGGAGCTGTGACCCTCTCNAACTTCCAGGGCAAAGTGATGATGACAGTCAATGCAACCGATGTTACTGACGTGATTACCATCCCAACAGCTGCTGGGAAAAACTTGTGCATTGTGAGGGCTATGGACGTGGGATACCTTTGTGAGGATACTATCACTTATGAATGTCCGGTCCTAGCTGCTGGAAATGACCCTGAAGACATTGACTGCTGGTGCACGAAATCATCTGTTTACGTGCGCTATGGAAGATGCACAAAAACTCGCCATTCTCGTCGAAGCAGAAGGTCTCTGACTGTCCAGACACATGGAGAAAGCACACTGGCCAACAAGAAAGGAGCTTGGTTGGACAGCACAAAAGCCACGAGGTATCTGGTGAAGACAGAATCATGGATACTGAGAAACCCGGGCTACGCCCTCGTTGCAGCGATCATTGGATGGATGCTAGGAAGCAACACAATGCAACGCGTCGTGTTTGCCATCCTATTGCTCCTGGTGGCACCAGCATACAGCTTCAATTGCTTGGGAATGAGTAACAGAGACTTCCTGGAGGGAGTGTCTGGAGCTACATGGGTTGATCTGGTACTGGAAGGCGATAGTTGTGTGACCATAATGTCAAAGGACAAGCCAACCATTGATGTTAAAATGATGAACATGGAAGCAACCAACCTCGCGGATGTGCGCAGCTACTGTTACCTAGCTTCGGTCAGTGACTTGTCAACGAGAGCCGCGTGTCCAACCATGGGTGAAGCCCACAACGAGAAAAGAGCTGACCCCGCCTTCGTTTGCAAGCAAGGCGTTGTGGACAGAGGATGGGGAAATGGCTGCGGACTGTTTGGAAAGGGAAGCATTGACACATGCGCGAAGTTTGCCTGTACAACCAAAGCAACTGGATGGATCATCCAGAAGGAGAACATCAGGTATGAGGTCGCCATATTTGTGCATGGCCCGACGACTGTTGAATCTCATGGCAATTATTCAACACAGATGGGGGCCACCCAGGCTGGAAGATTCAGTATAACTCCATCGGCGCCATCTTACACGCTAAAGTTGGGTGAGTATGGTGAGGTCACGGTTGACTGTGAGCCACGGTCAGGAATAGACACTAGCGCCTATTACGTTATGTCAGTTGGTGCGAAGTCCTTCCTGGTTCACCGAGAATGGTTTATGGATCTGAACCTGCCATGGAGCAGTGCTGGAAGCACCACGTGGAGGAATCGGGAAACACTGATGGAGTTTGAAGAACCTCATGCCACCAAACAATCTGTTGTGGCTCTAGGGTCGCAGGAAGGTGCATTGCACCAAGCTCTGGCCGGAGCGATTCCTGTTGAGTTCTCAAGCAGTACTGTGAAGTTGACATCAGGACATCTGAAGTGCAGGGTGAAGATGGAGAAGTTGCAGCTTAAGGGAACAACATATGGAGTATGTTCAAAAGCGTTTAAATTCGCTGGGACTCCCGCTGACACTGGCCATGGAACGGTGGTGTTGGAACTGCAATACACCGGAACAGACGGTCCCTGCAAAGTGCCCATTTCTTCCGTAGCTTCTCTGAATGACCTCACACCTGTTGGAAGACTGGTGACCGTGAATCCATTTGTGTCTGTGGCCACAGCCAACTCGAAGGTCTTGATTGAACTCGAACCCCCGTTTGGTGACTCTTACATCGTGGTGGGAAGAGGAGAACAGCAGATAAACCACCACTGGCACAAATCTGGGAGCAGCATCGGAAAGGCCTTTACCACTACACTCAGAGGAGCTCAACGACTCGCAGCTCTTGGAGACACTGCTTGGGATTTTGGGTCAGTTGGAGGGGTTTTCACCTCAGTGGGGAAAGCCATACACCAAGTCTTTGGAGGAGCTTTTAGATCACTCTTTGGAGGGATGTCCTGGATCACACAGGGACTTTTAGGAGCCCTTCTGTTGTGGATGGGAATCAATGCCCGTGACAGGTCAATTGCCATGACGTTTCTTGCGGTTGGAGGAGTTTTGCTCTTCCTTTCGGTCAACGTCCATGCTGACACAGGCTGTGCCATTGATATTGGCAGGCAAGAGCTCCGGTGTGGAAGTGGAGTGTTTATCCACAACGATGTGGAAGCCTGGATGGATCGTTACAAGTTCTACCCGGAGACACCACAAGGCCTAGCAAGAATCATTCAGAAAGCACATGCAGAAGGAGTCTGCGGCTTGCGTTCCGTTTCCAGACTCGAGCACCAAATGTGGGAAGCTATTAAGGATGAGCTGAACACCCTGTTGAAAGAGAATGGAGTCGACTTGAGTGTCGTGGTGGAAAAACAAAATGGGATGTATAAAGCAGCACCAAAACGTTTGGCTGCCACCACCGAAAAACTGGAGATGGGTTGGAAGGCTTGGGGCAAGAGCATCATCTTTGCTCCAGAACTAGCTAACAACACCTTTGTCATCGACGGTCCTGAGACTGAGGAATGCCCAACGGCCAACCGAGCATGGAACAGTATGGAGGTAGAAGACTTTGGATTTGGATTGACAAGCACTCGCATGTTCCTGAGGATTCGGGAAACGAACACAACTGAATGCGACTCGAAGATCATAGGAACCGCCGTCAAGAACAACATGGCTGTGCATAGTGATCTGTCGTACTGGATAGAGAGCGGACTCAACGACACCTGGAAGCTTGAGAGGGCNGTTCTAGGAGAAGTCAAATCATGCACCTGGCCAGAAACCCACACTTTGTGGGGTGATGGAGTTCTGGAAAGTGATCTCATCATACCCATCACCTTGGCAGGACCCAGAAGCAATCATAACAGGAGACCAGGGTACAAAACTCAGAACCAAGGTCCATGGGATGAGGGGCGTGTCGAGATTGACTTTGACTATTGCCCAGGAACAACAGTAACTATAAGTGANAGTTGCGGACACCGTGGACCTGCGGCACGCACAACCACTGAGAGTGGGAAGCTCATTACAGACTGGTGCTGCAGAAGCTGCACCCTCCCTCCACTGCGCTTCCAGACTGAAAATGGCTGTTGGTACGGAATGGAAATTCGACCCACGCGGCACGACGAAAAGACCCTCGTGCAATCGAGAGTGAATGCATACAACGCCGACATGATTGATCCTTTTCAGTTGGGCCTTCTGGTCGTGTTCTTGGCCACCCAGGAGGTCCTTCGCAAGAGGTGGACGGCCAAGATCAGCATTCCAGCTATCATGCTTGCACTCCTAGTCCTAGTGTTTGGGGGTATTACGTACACTGATGTCCTGCGATATGTCATTCTCGTCGGCGCCGCGTTTGCTGAAGCAAACTCAGGAGGAGACGTTGTGCACTTGGCACTCATGGCCACATTCAAGATTCAACCAGTCTTTCTGGTGGCTTCCTTTTTGAAGGCAAGGTGGACCAACCAAGAGAGTATTTTGCTCATGCTTGCAGCTGCTTTCTTTCAAATGGCTTACTATGACGCCAAGAATGTTTTGTCATGGGAAGTGCCTGACGTTTTGAACTCTCTCTCCGTCGCGTGGATGATTCTCAGAGCTATAAGCTTCACCAACACNTCAAATGTGGTGGTGCCGCTGCTGGCCCTTCTGACACCTGGACTAAAATGCCTAAACCTTGATGTGTACAGAATTTTGCTACTCATGGTTGGAGTTGGAAGCCTCATCAAAGAAAAAGGAGCTCTGCAGCAAAAAAGAAAGGGGCTTGCCTCATCTGCCTAGCGCTGGCGTCTACAGGAGCGTTTAATCCAATGATACTTGCAGCTGGGCTGATGGCTTGCGACCCCAACCGCAAGCGGGGCTGGCCTGCCACAGAAGTGATGACTGCAGTTGGACTCATGTTTGCCATCGTTGGGGGTCTGGCAGAACTTGACATAGACTCTATGGCTATCCCCATGACCATTGCCGGACTCATGTTCGTGGCATTTGTCATCTCTGGAAAGTCAACAGACATGTGGATTGAGAGGACGGCTGATATTACTTGGGAGAGTGACGCTGAAATCACAGGCTCTAGTGAAAGAGTAGATGTTAGGCTGGATGATGATGGAAATTTTCAATTGATAAATGACCCTGGGGCGCCATGGAAAATTTGGATGCTTAGGATGGCCTGCCTGGCAATAAGTGCCTACACACCTTGGGCGATTCTTCCCTCGGTCATTGGATTCTGGATAACCCTTCAGTACACAAAGAGAGGAGGTGTCCTTTGGGACACACCATCACCCAGGGAGTACAAGAAGGGTGATACCACCACTGGCGTTTACAGAATCATGACTCGAGGTTTGCTTGGCAGTTACCAAGCTGGAGCCGGAGTGATGGTAGAGGGAGTGTTCCACACACTATGGCACACCACTAAGGGAGCTGCTCTCATGAGTGGTGAAGGACGTCTGGATCCCTATTGGGGGAGCGTGAAAGAAGACCGGCTTTGCTATGGGGGGCCATGGAAACTCCAACATAAGTGGAATGGACATGATGAGGTCCAAATGATTGTTGTGGAGCCAGGAAAAAATGTGAAAAACGTCCAGACTAAGCCCGGAGTGTTTAAGACACCAGAAGGAGAAATTGGGGCAGTTACGCTAGACTATCCCACTGGAACGTCAGGTTCCCCCATTGTGGATAAAAATGGAGATGTGATTGGATTGTATGGGAACGGCGTCATCATGCCTAATGGTTCATACATAAGCGCCATTGTGCAAGGAGAGAGAATGGAAGAACCGGCACCAGCTGGCTTCGAACCTGAAATGTTGAGGAAGAAACAGATCACTGTCCTTGATCTGCACCCCGGAGCAGGAAAGACGCGCAAGATACTTCCCCAAATCATCAAGGAGGCCATCAACAAAAGANTGAGGACGGCTGTACTGGCACCCACCAGGGTCGTTGCTGCTGAGATGTCTGAGGCCCTGAGAGGACTTCCCATCCGGTACCAAACCTCAGCGGTGCACAGAGAGCACAGTGGAAATGAGATCGTTGATGTCATGTGCCATGCCACTCTCACACACAGGCTGATGTCTCCACACAGAGTCCCCAATTACAATCTGTTCATAATGGATGAAGCCCATTTCACGGATCCAGCGAGCATCGCAGCCAGAGGATACATAGCAACCAAGGTTGAATTGGGCGAAGCCGCCGCGATTTTCATGACGGCAACGCCACCCGGGACTTCTGACCCCTTTCCAGAGTCCAATGCTCCTATCTCGGACATGCAAACAGAGATCCCAGACAGAGCCTGGAACACTGGATATGAATGGATAACTGAGTATGTTGGAAAGACCGTTTGGTTTGTTCCAAGTGTGAGAATGGGAAATGAAATTGCCCTCTGTCTGCAACGGGCGGGGAAGAAGGTTATCCAGCTGAACAGAAAGTCTTATGAGACAGAGTACCCCAAGTGCAAGAACGATGATTGGGATTTTGTCATCACCACAGACATATCAGAAATGGGAGCAAACTTCAAGGCAAGCAGAGTGATCGACAGCCGCAAAAGCGTGAAACCCACCATCATTGAGGAAGGTGATGGAAGAGTCATCCTGGGGGAACCCTCAGCCATCACGGCTGCCAGCGCTGCTCAGCGGAGAGGACGCATAGGAAGAAACCCATCACAAGTTGGTGATGAGTATTGCTATGGAGGGCACACAAATGAGGATGATTCCAACTTTGCCCACTGGACAGAGGCTCGCATCATGCTAGACAACATCAACATGCCGAATGGTCTGGTGGCCCAACTATATCAGCCTGAGCGCGAGAAGGTGTACACCATGGACGGGGAATATAGGCTCAGAGGGGAAGAACGGAAGAACTTCCTTGAATTCCTGAGAACAGCTGATTTACCAGTCTGGCTCGCTTACAAAGTGGCAGCAGCAGGAATATCATACCATGACCGGAAGTGGTGCTTTGATGGACCTCGAACCAACACGATTCTTGAAGACAACAATGAAGTTGAAGTCATCACGAAGTTGGGTGAGAGAAAGATCTTAAGACCCAGGTGGGCGGATGCCAGAGTGTACTCAGACCACCAAGCTCTAAAGTCCTTCAAAGATTTTGCATCAGGGAAACGATCACAAATCGGGCTCGTTGAGGTGCTCGGGAGAATGCCTGAGCACTTCATGGGGAAAACTTGGGAGGCGTTGGACACGATGTATGTGGTGGCAACCGCTGAAAAAGGAGGCCGAGCTCACAGGATGGCTCTTGAGGAGCTACCGGACGCCCTTCAGACAATAGCTTTGATTGCACTATTGAGTGTGATGTCATTAGGTGTGTTTTTTCTACTCATGCAAAGGAAAGGCATTGGTAAGATTGGCTTGGGAGGAGTGATCTTAGGAGCTGCCACATTCTTCTGCTGGATGGCTGAAGTCCCAGGAACGAAAATAGCAGGCATGCTCCTGCTTTCCCTGCTGCTCATGATCGTTTTGATTCCGGAGCCGGAAAAGCAGCGCTCACAGACTGACAACCAGCTTGCCGTGTTCTTGATTTGCGTGCTCACACTGGTCAGCGCCGTGGCTGCCAATGAAATGGGTTGGCTGGACAAGACCAAGAATGACATCAGCAGCCTGTTGGGACACAAGCCAGAAACTAGAGAGACGACNCTGGGAGTTGAAAACTTTTTGCTTGATCTGCGGCCTGCTACAGCATGGTCGCTCTATGCCGTAACGACAGCCGTTCTTACCCCTTTGCTGAAACATCTAATCACGTCAGACTACATCAACACTTCGCTGACCTCAATAAACGTCCAAGCCAGTGCGTTGTTCACCTTGGCCAGAGGCTTTCCTTTTGTGGACGTTGGTGTGTCAGCTCTCTTGCTGGCGGCCGGGTGCTGGGGCCAAGTGACCCTGACTGTGACTGTGACTGCAGCTGCCCTGCTCTTTTGCCACTATGCTTACATGGTACCAGGCTGGCAAGCGGAAGCCATGCGATCCGCCCAGCGGCGGACAGCTGCTGGCATCATGAAAAATGCAGTGGTGGATGGGATCGTGGCTACCGATGTACCTGAACTTGAGCGAACAACTCCAGTCATGCAGAAAAANGTTGGACAGATCATGCTGATCTTGGTGTCAATGGCCGCGGTTGTCGTCAATCCATCAGTGAGAACTGTCAGAGAGGCTGGAATTCTGACTACAGCAGCAGCAGTCACCTTATGGGAGAATGGTGCTAGTTCAGTGTGGAATGCAACGACAGCTATTGGGCTTTGTCACATCATGCGAGGAGGATGGCTCTCGTGTCTCTCCATCACGTGGACTCTCATCAAAAACATGGAAAAACCAGGCCTCAAGAGGGGTGGAGCCAAGGGACGCACGCTAGGGGAAGTTTGGAAGGAGAGACTCAACCACATGACGAAGGAAGAATTTACCAGATACAGAAAAGAAGCCATCACTGAAGTCGACNGCTCCGCGGCAAAACATGCCAGGAGAGAGGGAAACATCACTGGAGGCCATCCAGTCTCACGGGGAACCGCGAAATTACGGTGGTTGGTGGAAAGGCGTTTCCTCGAGCCAGTGGGAAAGGTTGTGGATCTCGGATGTGGTAGAGGCGGCTGGTGTTATTACATGGCTACCCAGAAGAGGGTACAGGAAGTGAAAGGGTACACGAAAGGAGGACCTGGCCATGAAGAGCCACAACTGGTGCAGAGCTATGGTTGGAATATTGTTACCATGAAGAGCGGAGTCGACGTCTTCTACAGACCATCAGAAGCGAGCGACACACTGCTCTGTGACATTGGAGAGTCATCGTCAAGTGCCGAGGTAGAAGAACACCGCACNGTCCGTGTCTTGGAGATGGTGGAAGACTGGCTGCACAGAGGACCTAAGGAATTCTGCATCAAAGTGCTATGCCCTTACATGCCCAGAGTGATTGAGAAGATGGAAACACTCCAAAGGCGATATGGAGGTGGCCTTGTGAGAAACCCCCTTTCACGCAACTCTACCCATGAGATGTACTGGGTGAGCCACGCTTCAGGCAACATTGTCCACTCCGTGAACATGACAAGCCAGGTGCTTCTGGGGAGGATGGAAAAGAAAACATGGAAGGGACCCCAGTTTGAGGAAGATGTNAACTTGGGAAGTGGAACNNGGGCAGTAGGGAAGCCTCTCCTCAATTCTGATACTAGCAAGATCAAGAGCCGAATTGAGAGGCTGAAGAAAGAATACAGCTCCACATGGCACCAGGATGCGAATCACCCCTACAGGACCTGGAACTACCACGGAAGCTATGAAGTGAAACCAACCGGCTCAGCCAGCTCCCTTGTGAATGGGGTAGTTAGATTACTCTCAAAACCATGGGACACTATCACCAATGTGACCACGATGGCCATGACAGACACCACTCCTTTCGGTCAACAACGAGTGTTCAAGGAAAAGGTGGATACAAAGGCTCCAGAGCCTCCAGAAGGGGTCAAATACGTCCTCAATGAGACCACGAACTGGCTGTGGGCTTTCCTAGCCCGCGATAAGAAACCCAGGATGTGTTCCCGGGAGGAATTCATTGGAAAAGTCAACAGTAATGCCGCCCTGGGAGCGATGTTTGAAGAACAGNACCTGGAAGAACGCCCGGGAAGCCGTGGAGGATCCAAAGTTTTGGGAGATGGTGGATGAGGAGCGTGAGGCGCATCTCCGTGGAGAATGCAACACCTGCATCTACAACATGATGGGAAAGAGAGAGAAGAAGCCTGGAGAGTTCGGCAAAGCTAAAGGCAGCAGAGCCATTTGGTTCATGTGGCTAGGGGCCCGCTTCCTGGAGTTTGAAGCTCTCGGATTCCTCAATGAAGACCACTGGCTGGGTAGGAAGAACTCAGGAGGAGGAGTTGAAGGCTTAGGACTGCAGAAGCTTGGGTACATCTTGAAGGAAGTCGGAACAAAGCCTGGAGGAAAGATCTATGCCGATGATACCGCAGGCTGGGACACACGCATCACCAAAGCTGACCTCGAGAACGAAGCGAAGGTTCTTGAACTGCTGGACGGAGAACACCGACGCCTAGCGCGGTCCATCATTGAGCTCACATACCGACACAAAGTCGTGAAAGTGATGAGGCCAGCGGCCGACGGGAAAACTGTGATGGATGTCATCTCCAGAGAGGATCAGAGAGGAAGCGGGCAGGTAGTGACTTACGCCCTGAACACCTTCACTAATCTAGCAGTTCAGCTGGTCAGAATGATGGAGGGGGAGGGGGTCATTGGACCTGACGATGTTGAAAAACTGGGAAAAGGAAAAGGCCCTAAGGTCAGAACCTGGCTGTTTGAGAATGGNGAGGAGCGTCTCAGTCGCATGGCCGTCAGCGGTGATGACTGCGTGGTGAAACCTTTGGACGACCGCTTCGCCACATCACTACACTTCCTAAATGCCATGTCAAAGGTCCGCAAGGACATCCAGGAATGGAAACCCTCGACAGGGTGGTATGACTGGCAGCAGGTCCCATTCTGTTCAAACCATTTCACGGAACTGATCATGAAGGATGGCAGGACGTTGGTGGTCCCGTGTCGTGGACAAGACGAGTTGATTGGACGGGCCAGAATCTCTCCAGGGGCTGGATGGAATGTGCGCGACACCGCCTGTCTGGCGAAGTCATACGCGCAGATGTGGCTGTTGCTTTATTTTCACCGTAGAGACCTGAGATTGATGGCTAACGCCATCTGTTCCGCTGTGCCTGTCAACTGGGTTCCCACAGGGCGTACCACCTGGTCGATCCACGCAAAAGGAGAATGGATGACGACAGAAGACATGCTCGCAGTCTGGAACAGAGTGTGGATTGAGGAGAATGAGTGGATGGAAGACAAAACACCAGTTGAGAGGTGGAGTGATGTTCCATACTCTGGAAAGAGAGAAGACATTTGGTGTGGCAGTTTGATTGGCACACGAACCCGCGCTACTTGGGCTGAAAATATCCATGTGGCAATCAATCAGGTCCGTTCGGTGATTGGAGAAGAGAAGTATGTGGATTACATGAGCTCTCTGAGGAGGTATGAAGACACCATTGTAGTTGAGGACACTGTTCTGTAAATAATAAAGCTGTATTGAGTAGTTGTACAGTTGTAGTGTTTATAGCAATTTGAATTATGATTAATTACTTAGGCTTAAGATAGTATTATAGTTAGTTTAGTGTAAATAGGTTTTATTGAGAATGGAAGTCAGGCCAGATTAATGCTGCCACCGGAAGTTGAGTAGACGAGGTGCTGCCTGCGGCTCAACCCCAGGAGGACTGGGTGACCAAAGCTGCGAGGTGATCCACGTAAGCCCTCAGAACCGTCTCGGAAGGAGGACCCCACGTGCTTTAGCCTCAAAGCCCAGTGTCAGACCACACTCTAGTGTGCCACTCTGCGGAGAGTGCAGTCTGCGATAGTGCCCCAGGTGGACTGGGTTAACAAAGGCAAAACATCGCCCCACGCGGCCATAACCCTGGCTATGGTGTTAACCAGGGAGAAGGGACTAGAGGTTAGAGGAGACCCCGCGTCAAAAAGTGCACGGCCCAACTTGGCTGAAGCTGTAAGCCAAGGGAAGGACTAGAGGTTAGAGGAGACCCCGTGCCAAAAACACCAAAAGAAACAGCATATTGACACCTGGGATAGACTAGGGGATCTTCTGCTCTGCACAACCAGCCACACGGCACAGTGCGCNNN

Consensus genome Sample 3:

>sample3
ACAAACTTAGTAGTGTTTGTGAGGATTAATAACAATTAACACAGTGCGAGCTGTTTCTTAGCACGAAGATCTCGATGTCTAAGAAACCAGGAGGGCCCGGTAAAAACCGGGCTGTCAATATGCTAAAACGCGGTATGCCCCGCGGATTGTCCTTGATAGGACTGAAGAGGGCTATGCTGAGTCTGATTGACGGGAAAGGCCCAATACGTTTCGTGTTGGCTCTTTTGGCGTTTTTCAGATTCACTGCAATCGCTCCGACTCGTGCGGTGCTGGACAGATGGAGAGGCGTCAACAAACAAACAGCGATGAAGCATCTCTTGAGTTTCAAGAAAGAACTAGGAACTCTGACCAGTGCCATCAACCGCCGGAGCACAAAACAAAAGAAAAGAGGAGGCACAGCGGGCTTTACTATTTTGCTTGGGCTGATCGCTTGTGCTGGAGCTGTGACCCTCTCGAACTTCCAGGGCAAAGTGATGATGACAGTCAATGCAACCGATGTTACTGACGTGATTACCATCCCAACAGCTGCTGGGAAAAACCTGTGCATTGTGAGGGCTATGGACGTGGGATACCTTTGTGAGGATACTATCACTTATGAATGTCCGGTCCTAGCTGCTGGAAATGACCCTGAAGACATTGACTGCTGGTGCACGAAATCATCTGTTTACGTGCGCTATGGAAGATGCACAAAAACTCGCCATTCCCGTCGAAGCAGAAGGTCTCTGACTGTCCAGACACATGGAGAAAGCACACTGGCCAACAAGAAAGGAGCTTGGTTGGACAGCACAAAAGCCACGAGGTATCTGGTGAAGACAGAATCATGGATACTGAGAAACCCGGGCTACGCCCTCGTTGCAGCGATCATTGGATGGATGCTAGGAAGCAACACAATGCAACGCGTCGTGTTTGCCATCCTATTGCTCCTGGTGGCACCAGCATACAGCTTCAATTGCTTGGGAATGAGTAACAGAGACTTCCTGGAGGGAGTGTCTGGAGCTACATGGGTTGATCTGGTACTGGAAGGCGATAGTTGTGTGACCATAATGTCAAAGGACAAGCCAACCATTGATGTTAAAATGATGAACATGGAAGCAACCAACCTCGCGGATGTGCGCAGCTACTGTTACCTAGCTTCGGTCAGTGACTTGTCAACGAGAGCNGNGTGTCCAACCATGGGTGAAGCCCACAACGAGAAAAGAGCTGACCCCGCCTTCGTTTGCAAGCAAGGCGTTGTGGACAGAGGATGGGGAAATGGCTGCGGACTGTTTGGAAAGGGAAGCATTGACACATGCGCGAAGTTTGCCTGTACAACCAAAGCAACTGGATGGATCATCCAGAAGGAGAACATCAAGTATGAGGTCGCCATATTTGTGCATGGCCCGACGACTGTTGAATCTCATGGCAATTATTCAACACAGATGGGGGCCACCCAGGCTGGAAGATTCAGTATAACTCCATCGGCGCCATCTTACACGCTAAAGTTGGGTGAGTATGGTGAGGTCACGGTTGACTGTGAGCCACGGTCAGGAATAGACACTAGCGCCTATTACGTTATGTCAGTTGGTGCGAAGTCCTTCCTGGTTCACCGAGAATGGTTTATGGATCTGAACCTGCCATGGAGCAGTGCTGGAAGCACCACGTGGAGGAATCGGGAAACACTGATGGAGTTTGAAGAACCTCATGCCACCAAACAATCTGTTGTGGCTCTAGGGTCGCAGGAAGGTGCATTGCACCAAGCTCTGGCCGGAGCGATTCCTGTTGAGTTCTCAAGCAATACTGTGAAGTTGACATCAGGACATCTGAAGTGCAGGGTGAAGATGGAGAAGTTGCAGCTTAAGGGAACAACATATGGAGTATGTTCAAAAGCGTTTAAATTCGCTGGGACTCCCGCTGACACTGGCCATGGAACGGTGGTGTTGGAACTGCAATACACCGGAACAGACGGTCCCTGCAAAGTGCCCATTTCTTCCGTAGCTTCTCTGAATGACCTCACACCTGTTGGAAGACTGGTGACCGTGAATCCATTTGTGTCTGTGGCCACAGCCAACTCGAAGGTCTTGATTGAACTCGAACCCCCGTTTGGTGACTCTTACATCGTGGTGGGAAGAGGAGAACAGCAGATAAACCACCACTGGCACAAATCTGGGAGCAGCATCGGAAAGGCCTTTACCACTACACTCAGAGGAGCTCAACGACTCGCAGCTCTTGGAGACACTGCTTGGGATTTTGGGTCAGTTGGAGGGGTTTTCACNTCAGTGGGGAAAGCCATACACCAAGTCTTTGGAGGAGCTTTTAGATCACTCTTTGGAGGGATGTCCTGGATCACACAGGGACTTTTAGGAGCCCTTCTGTTGTGGATGGGAATCAATGCCCGTGACAGGTCAATTGCCATGACGTTTCTTGCGGTTGGAGGAGTTTTGCTCTTCCTTTCGGTCAACGTCCATGCTGACACAGGCTGTGCCATTGATATTGGCAGGCAAGAGCTCCGGTGTGGAAGTGGAGTGTTTATCCACAACGATGTGGAAGCCTGGATGGATCGTTACAAGTTCTACCCGGAGACACCACAAGGCCTAGCAAGAATCATTCAGAAAGCACATGCAGAAGGAGTCTGCGGCTTGCGTTCCGTTTCCAGACTCGAGCACCAAATGTGGGAAGCTATTAAGGATGAGCTGAACACCCTGTTGAAAGAGAATGGAGTCGACTTGAGTGTCGTGGTGGAAAAACAAAATGGGATGTATAAAGCAGCACCAAAACGTTTGGCTGCCACCACCGAAAAACTGGAGATGGGTTGGAAGGCTTGGGGCAAGAGCATCATCTTTGCTCCAGAACTAGCTAACAACACCTTTGTCATCGACGGTCCTGAGACTGAGGAATGCCCAACGGCCAACCGAGCATGGAACAGTATGGAGGTAGAAGACTTTGGATTTGGATTGACAAGCACTCGCATGTTCCTAAGGATTCGGGAAACGAACACAACTGAATGCGACTCGAAGATCATAGGAACCGCCGTCAAGAACAACATGGCTGTGCATAGTGATCTGTCGTACTGGATAGAGAGCGGACTCAACGACACCTGGAAGCTTGANAGGGCNGTTCTAGGAGAAGTCAAATCATGCACCTGGCCAGAAACCCACACTTTGTGGGGTGATGGAGTTCTGGAAAGTGATCTCATCATACCCATCACCTTGGCAGGACCCAGAAGCAATCATAACAGGAGACCAGGGTACAAAACTCAGAACCAAGGTCCATGGGATGAGGGGCGTGTCGAGATTGACTTTGACTATTGCCCAGGAACAACAGTAACTATAAGTGATAGTTGCGGACACCGTGGACCTGCGGCACGCACAACCACTGAGAGTGGGAAGCTCATTACAGACTGGTGCTGCAGAAGCTGCACCCTCCCTCCACTGCGCTTCCAGACTGAAAATGGCTGTTGGTACGGAATGGAAATTCGACCCACGCGGCACGACGAAAAGACCCTCGTGCAATCGAGAGTGAATGCATACAACGCCGACATGATTGATCCTTTTCAGTTGGGCCTTCTGGTCGTGTTCTTGGCCACCCAGGAGGTCCTTCGCAAGAGGTGGACGGCCAAGATCAGCATTCCAGCTATCATGCTTGCACTCCTAGTCCTAGTGTTTGGGGGTATTACGTACACTGATGTCCTGCGATATGTCATTCTCGTCGGCGCCGCGTTTGCTGAAGCAAACTCAGGAGGAGACGTTGTGCACTTGGCACTCATGGCCACATTCAAGATTCAACCAGTCTTTCTGGTGGCTTCCTTTTTGAAGGCAAGGTGGACCAACCAAGAGAGTATTTTGCTCATGCTTGCAGCTGCTTTCTTTCAAATGGCTTACTATGANGCCAAGAATGTTTTGTCATGGGAAGTGCCTGACGTTTTGAACTCTCTCTCCGTCGCNTGGATGATTCTCAGAGCTATAAGCTTCACCAGCACCTCAAATGTGGTGGTGCCGCTGCTGGCCCTTCTGACACCTGGACTAAAATGCCTAAACCTTGATGTGTACAGAATTTTGCTACTCATGGTTGGAGTTGGAAGCCTCATCAAAGAAAAAAGGAGCTCTGCAGCAAAAAAGAAAGGGGCTTGCCTCATCTGCCTAGCGCTGGCGTCTACAGGAGCGTTCAATCCAATGATACTTGCAGCTGGGCTGATGGCTTGCGACCCCAACCGCAAGCGGGGCTGGCCTGCCACAGAAGTGATGACTGCAGTTGGACTCATGTTTGCCATCGTTGGGGGTCTGGCAGAACTTGACATAGACTCTATGGCTATCCCCATGACCATTGCCGGACTCATGTTCGTGGCATTTGTCATCTCTGGAAAGTCAACAGACATGTGGATTGAGAGGACGGCTGATATTACTTGGGAGAGTGACGCTGAAATCACAGGCTCTAGTGAAAGAGTAGATGTTAGGCTGGATGATGATGGAAATTTTCAATTGATAAATGACCCTGGGGCGCCATGGAAAATTTGGATGCTTAGGATGGCCTGCCTGGCAATAAGTGCCTACACACCTTGGGCGATTCTTCCCTCGGTCATTGGATTCTGGATAACCCTTCAGTACACAAAGAGAGGAGGTGTCCTTTGGGACACACCATCACCCAGGGAGTACAAGAAGGGTGATACCACCACTGGCGTTTACAGAATCATGACTCGAGGTTTGCTTGGCAGTTACCAAGCTGGAGCCGGAGTGATGGTAGAGGGAGTGTTCCACACACTATGGCACACCACTAAGGGAGCTGCTCTCATGAGTGGTGAAGGACGTCTGGATCCCTATTGGGGGAGCGTGAAAGAAGACCGGCTTTGCTATGGGGGGCCATGGAAACTCCAACATAAATGGAATGGACATGATGAGGTCCAAATGATTGTTGTGGAGCCAGGAAAAAATGTGAAAAACGTCCAGACTAAGCCCGGAGTGTTTAAGACACCAGAAGGAGAAATTGGGGCAGTTACGCTAGACTATCCCACTGGAACGTCAGGTTCCCCCATTGTGGATAAAAATGGAGATGTGATTGGATTGTATGGGAACGGCGTCATNATGCCTAATGGTTCATACATAAGCGCCATTGTGCAAGGAGAGAGAATGGAAGAACCGGCACCAGCTGGCTTCGAACCTGAAATGTTGAGGAAGAAACAGATTACTGTCCTTGATCTGCACCCCGGAGCAGGAAAGACGCGCAAGATACTTCCCCAAATCATCAAGGAGGCCATCAACAAAAGANTGAGGACGGCTGTACTGGCACCCACCAGGGTCGTTGCTGCTGAGATGTCTGAGGCCCTGAGAGGACTTCCCATCCGGTACCAAACCTCAGCAGTGCACAGAGAGCACAGTGGAAATGAGATCGTTGATGTCATGTGCCATGCCACTCTCACACACAGGCTGATGTCTCCACACAGAGTCCCCAATTACAATCTGTTCATAATGGATGAAGCCCATTTCACGGATCCAGCGAGNATCGCAGCCAGAGGATACATAGCAACCAAGGTTGAATTGGGCGAAGCCGCCGCGATTTTCATGACGGCAACGCCACCCGGGACTTCTGACCCCTTTCCAGAGTCCAATGCTCCTATCTCGGANATGCAAACAGAGATCCCAGACAGAGCCTGGAACACTGGATATGAATGGATAACTGAGTATGTTGGAAAGACCGTTTGGTTTGTTCCAAGTGTGAAAATGGGAAATGAAATTGCCCTCTGTCTGCAACGGGCGGGGAAGAAGGTTATCCAGCTGAACAGAAAGTCCTATGAGACAGAGTACCCCAAGTGCAAGAACGATGATTGGGATTTTGTCATCACCACAGACATATCAGAAATGGGAGCAAACTTCAAGGCAAGCAGAGTGATCGACAGCCGCAAAAGCGTGAAACCCACCATCATTGAGGAAGGTGATGGAAGAGTCATNCTGGGGGAACCCTCAGCCATCACGGCTGCCAGCGCTGCTCAGCGGAGAGGACGCATAGGAAGAAACCCATCACAAGTTGGTGATGAGTATTGCTATGGAGGGCACACAAATGAGGATGATTCCAACTTTGCCCACTGGACAGAGGCTCGCATCATGCTAGACAACATCAACATGCCGAATGGTCTGGTGGCCCAACTATATCAGCCTGAGCGCGAGAAGGTGTACACCATGGACGGGGAATATAGGCTCAGAGGGGAAGAACGGAAGAACTTCCTTGAATTCCTGAGAACAGCTGATTTACCAGTCTGGCTCGCTTACAAAGTGGCAGCAGCAGGAATATCATACCATGACCGGAAGTGGTGCTTTGATGGACCTCGAACCAACACGATTCTTGAAGACAACAATGAAGTTGAAGTTATCACGAAGTTGGGTGAGAGAAAGATCTTAAGACCCAGGTGGGCGGATGCCAGAGTGTACTCAGACCACCAAGCTCTAAAGTCCTTCAAAGATTTTGCATCAGGGAAACGATCACAAATCGGGCTCGTTGAGGTGCTCGGGAGAATGCCTGAGCACTTCATGGGGAAAACTTGGGAGGCGTTGGACACGATGTATGTGGTGGCAACCGCTGAAAAAGGAGGCCGAGCTCACAGGATGGCTCTTGAGGAGCTACCGGACGCCCTTCAGACAATAGCTTTGATTGCACTATTGAGTGTGATGTCCTTAGGTGTGTTTTTTCTACTCATGCAAAGGAAAGGCATTGGTAAGATTGGCTTGGGAGGAGTGATCTTAGGAGCTGCCACATTCTTCTGCTGGATGGCTGAAGTCCCAGGAACGAAAATAGCAGGCATGCTCCTGCTTTCCCTGCTGCTCATGATCGTTTTGATTCCGGAGCCGGAAAAGCAGCGCTCACAGACTGACAACCAGCTTGCCGTGTTCTTGATTTGTGTGCTCACACTGGTCAGCGCCGTGGCTGCCAATGAAATGGGTTGGCTGGACAAGACCAAGAATGACATCAGCAGCCTGTTGGGACACAAGCCAGAAACTAGAGAGACGACCCTGGGAGTTGAGAACTTTTTGCTTGATCTGCGGCCTGCTACAGCATGGTCGCTCTATGCCGTAACGACAGCCGTTCTTACCCCTTTGCTGAAACATCTAATCACGTCAGATTACATCAACACTTCGCTGACCTCAATAAACGTCCAAGCCAGTGCGTTGTTCACCTTGGCCAGAGGCTTCCCTTTTGTGGACGTTGGTGTGTCAGCTCTCTTGCTGGCGGCCGGGTGCTGGGGCCAAGTGACCCTGACTGTGACTGTGACTGCAGCTGCCCTGCTCTTTTGCCACTATGCTTACATGGTACCAGGCTGGCAAGCGGAAGCCATGCGATCCGCCCAGCGGCGGACAGCTGCTGGCATCATGAAAAATGCAGTGGTGGATGGGATCGTGGCTACCGATGTACCTGAACTTGAGCGAACAACTCCAGTCATGCAGAAAAAAGTTGGACAGATCATGCTGATCTTGGTGTCAATGGCCGCGGTTGTCGTCAATCCATCAGTGAGAACTGTCAGAGAGGCTGGAATTCTGACTACAGCAGCAGCAGTCACCTTATGGGAGAATGGTGCTAGTTCAGTGTGGAATGCAACGACAGCTATTGGGCTTTGTCACATCATGCGAGGAGGATGGCTCTCGTGTCTCTCCATCACGTGGACTCTCATCAAAAACATGGAAAAACCAGGCCTCAAGAGGGGTGGAGCCAAGGGACGCACGCTAGGGGAAGTTTGGAAGGAGAGACTCAACCACATGACGAAGGAAGAATTTACCAGATACAGAAAAGAAGCCATCACTGAAGTCGACNGCTCCGCGGCAAAACATGCCAGGAGAGAGGGAAACATCACTGGAGGCCATCCAGTCTCACGGGGAACCGCGAAATTACGGTGGTTGGTGGAAAGGCGTTTCCTCGAGCCAGTGGGAAAGGTTGTGGATCTCGGATGTGGTAGAGGCGGCTGGTGTTATTACATGGCTACCCAGAAGAGGGTACAGGAAGTGAAAGGGTACACGAAAGGAGGACCTGGCCATGAAGAGCCACAACTGGTGCAGAGCTATGGTTGGAATATTGTTACCATGAAGAGCGGAGTCGACGTCTTCTACAGACCATCAGAAGCGAGCGACACACTGCTCTGTGACATTGGAGAGTCATCGTCAAGTGCCGAGGTAGAAGAACACCGCACCGTCCGTGTCTTGGAGATGGTGGAAGACTGGCTGCACAGAGGACCTAAGGAATTCTGCATCAAAGTGCTATGCCCTTACATGCCCAGAGTGATTGAGAAGATGGAAACACTCCAAAGGCGATATGGAGGTGGCCTTGTGAGAAACCCCCTTTCACGCAACTCTACCCATGAGATGTACTGGGTGAGCCACGCTTCAGGCAACATTGTCCACTCCGTGAACATGACGAGCCAGGTGCTTCTGGGGAGGATGGAAAAGAAAACATGGAAGGGACCCCAGTTTGAGGAAGATGTNAACTTGGGAAGTGGAACNNGGGCAGTAGGGAAGCCTCTCCTCAATTCTGATACTAGCAAGATCAAGAGCCGAATTGAGAGGCTGAAGAAAGAATACAGCTCCACATGGCACCAGGATGCGAATCACCCCTACAGGACCTGGAACTACCACGGAAGCTATGAAGTGAAACCAACCGGCTCAGCCAGCTCCCTTGTGAATGGGGTAGTTAGATTACTCTCAAAACCATGGGACACTATCACCAATGTGACCACGATGGCCATGACAGACACCACTCCTTTCGGTCAACAACGAGTGTTCAAGGAAAAGGTGGATACAAAGGCTCCAGAGCCTCCAGAAGGGGTCAAATACGTCCTCAATGAGACCACGAACTGGCTGTGGGCTTTTCTAGCCCGCGATAAGAAACCCAGGATGTGTTCCCGGGAGGAATTCATTGGAAAAGTCAACAGTAATGCCGCCCTGGGAGCGATGTTTGAAGAACAGNACCAATGGAAGAACGCCCGGGAAGCCGTGGAGGATCCAAAGTTTTGGGAGATGGTGGATGAGGAGCGTGAGGCGCATCTCCGTGGAGAATGCAACACCTGCATCTACAACATGATGGGAAAGAGAGAGAAGAAGCCTGGAGAGTTCGGCAAAGCTAAAGGCAGCAGAGCCATTTGGTTCATGTGGCTAGGGGCCCGCTTCCTGGAGTTTGAAGCTCTCGGATTCCTCAATGAAGACCACTGGCTGGGTAGGAAGAACTCAGGAGGAGGAGTTGAAGGCTTAGGACTGCAGAAGCTTGGGTACATCTTGAAGGAAGTCGGAACAAAGCCTGGAGGAAAGATCTATGCCGATGATACCGCAGGCTGGGACACACGCATCACCAAAGCTGACCTCGAGAACGAAGCGAAGGTTCTTGAACTGCTGGACGGAGAACACCGACGCCTAGCGCGGTCCATCATTGAGCTCACATACCGACACAAAGTCGTGAAAGTGATGAGGCCAGCGGCCGACGGGAAAACTGTGATGGATGTCATCTCCAGAGAGGATCAGAGAGGAAGCGGGCAGGTAGTGACTTACGCCCTGAACACCTTCACTAATCTAGCAGTTCAGCTGGTCAGAATGATGGAAGGGGAGGGGGTCATTGGACCTGACGATGTTGAAAAACTGGGAAAAGGAAAAGGCCCTAAGGTCAGAACCTGGCTGTTTGAGAATGGTGAGGAGCGTCTCAGTCGCATGGCCGTCAGCGGTGATGACTGCGTGGTGAAACCTTTGGACGACCGCTTCGCCACATCACTACACTTCCTAAATGCCATGTCAAAGGTCCGCAAGGACATCCAGGAATGGAAACCCTCGACAGGGTGGTATGACTGGCAGCAGGTCCCATTCTGTTCAAACCATTTCACGGAACTGATCATGAAGGATGGCAGGACGTTGGTGGTCCCGTGTCGTGGACAAGACGAGTTGATTGGACGGGCCAGAATCTCTCCAGGGGCTGGATGGAATGTGCGCGACACCGCCTGCCTGGCGAAGTCATACGCGCAGATGTGGCTGTTGCTTTNTTTTCACCGTAGAGACCTGAGATTGATGGCTAACGCCATCTGTTCCGCTGTGCCTGTCAACTGGGTTCCCACAGGGCGTACCACCTGGTCGATCCACGCAAAAGGAGAATGGATGACGACAGAAGACATGCTCGCAGTCTGGAACAGAGTGTGGATTGAGGAGAATGAGTGGATGGAAGACAAAACACCAGTTGAGAGGTGGAGTGATGTTCCATACTCTGGAAAGAGAGAAGACATTTGGTGTGGCAGTTTGATTGGCACACGAACCCGCGCTACTTGGGCTGAAAATATCCATGTGGCAATCAATCAGGTCCGTTCGGTGATTGGAGAAGAGAAGTATGTGGATTACATGAGCTCTCTGAGGAGGTATGAAGACACCATTGTAGTTGAGGACACTGTTTTGTAAATAATAAAGCTGTATTGAGTAGTTGTATAGTTGTAGTGTTTATAGCAATTTGAATTATGATTAATTATTTAGGCTTAAGATAGTATTATAGTTAGTTTAGTGTAAATAGGTTTTATTGAGAATGGAAGTCAGGCCAGATTAATGCTGCCACCGGAAGTTGAGTAGACGAGGTGCTGCCTGCGGCTCAACCCCAGGAGGACTGGGTGACCAAAGCTGCGAGGTGATCCACGTAAGCCCTCAGAACCGTCTCGGAAGGAGGACCCCACGTGCTTTAGCCTCAAAGCCCAGTGTCAGACCACACTCTAGTGTGCCACTCTGCGGAGAGTGCAGTCTGCGATAGTGCCCCAGGTGGACTGGGTTAACAAAGGCAAAACATCGCCCCACGCGGCCATAACCCTGGCTATGGTGTTAACCAGGGAGAAGGGACTAGAGGTTAGAGGAGACCCCGCGTCAAAAAGTGCACGGCCCAACTTGGCTGAAGCTGTAAGCCAAGGGAAGGACTAGAGGTTAGAGGAGACCCCGTGCCAAAAACACCAAAAGAAACAGCATATTGACACCTGGGATAGACTAGGGGATCTTCTGCTCTGCACAACCAGCCACACGGCACAGTGC

Consensus genome Sample 4:

>sample4
ACAAACTTAGTAGTGTTTGTGAGGATTAATAACAATTAACACAGTGCGAGCTGTTTCTTAGCACGAAGATCTCGATGTCTAAGAAACCAGGAGGGCCCGGTAAAAACCGGGCTGTCAATATGCTAAAACGCGGTATGCCCCGCGGATTGTCCTTGATAGGACTGAAGAGGGCTATGCTGAGTCTGATTGACGGGAAAGGCCCAATACGTTTCGTGTTGGCTCTTTTGGCGTTTTTCAGATTCACTGCAATCGCTCCGACTCGTGCGGTGCTGGACAGATGGAGAGGCGTCAACAAACAAACAGCGATGAAGCATCTCTTGAGTTTCAAGAAAGAACTAGGAACTCTGACCAGCGCCATCAACCGCCGGAGCACAAAACAAAAGAAAAGAGGAGGCACAGCGGGCTTTACTATCTTGCTTGGGCTGATCGCTTGTGCTGGAGCTGTGACCCTCTCGAACTTCCAGGGCAAAGTGATGATGACAGTCAATGCAACCGATGTTACTGACGTGATTACCATCCCAACAGCTGCTGGGAAAAACCTGTGCATTGTGAGGGCTATGGACGTGGGATACCTTTGTGAGGATACTATCACTTATGAATGTCCGGTCCTAGCTGCTGGAAATGACCCTGAAGACATTGACTGCTGGTGCACGAAATCATCTGTTTACGTGCGCTATGGAAGATGCACAAAAACTCGGCATTCCCGTCGAAGCAGAAGGTCTCTGACTGTCCAGACACATGGAGAAAGTACACTGGCCAACAAGAAAGGAGCTTGGTTGGACAGCACAAAAGCCACGAGATATCTGGTGAAGACAGAATCATGGATACTGAGAAACCCGGGCTACGCCCTCGTTGCAGCGGTCATTGGATGGATGCTAGGAAGCAACACAATGCAACGCGTCGTGTTTGCCATCCTATTGCTCCTGGTGGCACCAGCATACAGCTTCAATTGCTTGGGAATGAGTAACAGAGACTTCCTGGAGGGAGTGTCTGGAGCTACATGGGTTGATCTGGTACTGGAAGGCGATAGTTGTGTGACCATAATGTCAAAGGACAAGCCAACCATTGATGTNAAAATGATGAACATGGAAGCANCCAACCTCGCAGANGTGCGCAGCTACTGTTACCTAGCTTCGGTCAGTGACTTGTCAACGAGAGCNGCGTGTCCAACNATGGGTGAAGCCCACAACGAGAAAAGAGCTGACCCCGCCTTCGTTTGCAAGCAAGGCGTTGTGGACAGAGGATGGGGAAATGGCTGCGGACTGTTTGGAAAGGGAAGCATTGACACATGCGCGAAGTTTGCCTGTACAACCAAAGCAACTGGATGGATCATCCAGAAGGAGAACATCAAGTATGAGGTCGCCATATTTGTGCATGGCCCGACGACTGTTGAATCTCATGGCAATTATTCAACACAGGCAGGAGCCACCCAGGCTGGAAGANTCAGTATAACTCCATCGGCGCCATCTTACACGCTAAAGTTGGGTGAGTATGGTGAGGTCACGGTTGACTGTGAGCCACGGTCAGGAATAGACACTAGCGCCTATTACGTTATGTCAGTTGGTGCGAAGTCCTTCCTGGTTCACCGAGAGTGGTTTATGGATCTGAACCTGCCATGGAGCAGTGCTGGAAGCACCACGTGGAGGAATCGGGAAACACTGATGGAGTTTGAAGAACCTCATGCCACCAAACAATCTGTTGTGGCTCTAGGGTCGCAGGAAGGTGCATTGCACCAAGCTCTGGCCGGAGCGATTCCTGTTGAGTTCTCAAGCAATACTGTGAAGTTGACATCAGGACATCTGAAGTGTAGGGTGAAGATGGAGAAGTTGCAGCTTAAGGGAACAACATATGGAGTATGTTCAAAAGCGTTTAAATTCGCTGGGACTCCCGCTGACACTGGCCATGGAACGGTGGTGTTGGAACTGCAATACACCGGAACAGACGGTCCCTGCAAAGTGCCCATTTCTTCCGTAGCTTCNCTGAATGACCTCACACCTGTTGGAAGACTGGTGACCGTGAATCCATTTGTGTCTGTGGCCACAGCCAACTCGAAGGTCTTGATTGAACTCGAACCCCCGTTTGGTGACTCTTACATCGTGGTGGGAAGAGGAGAACAGCAGATAAACCACCACTGGCACAAATCTGGGAGCAGCATCGGAAAGGCCTTTACCACNACACTCAGAGGAGCTCAACGACTCGCAGCTCTTGGAGACACTGCTTGGGATTTTGGGTCAGTTGGAGGGGTTTTCACCTCAGTGGGGAAAGCCATACACCAAGTCTTTGGAGGAGCTTTTAGATCACTCTTTGGAGGGATGTCCTGGATNACACAGGGACTTNTAGGAGCCCTTCTGTTGTGGATGGGAATCAATGCCCGTGACAGGTCAATTGCCATGACGTTTCTTGCGGTTGGAGGAGTTTTGCTCTTCCTTTCGGTCAACGTCCATGCTGACACAGGCTGTGCCATTGATATTGGCAGGCAAGAGCTCCGGTGTGGAAGTGGAGTGTTTATCCACAACGATGTGGAAGCCTGGATGGATCGTTACAAGTTCTACCCGGAGACACCACAAGGCCTAGCAAGAATNATTCAGAAAGCACATGCAGAAGGAGTCTGCGGCTTGCGTTCCGTTTCCAGACTCGAGCACCAAATGTGGGAAGCNATTAAGGATGAGCTGAACACCCTGTTGAAAGAGAATGGAGTCGACTTGAGTGTCGTGGTGGAAAAACAAAATGGGATGTATAAAGCAGCACCAAAACGTTTGGCTGCCACCACCGAAAAACTGGAGATGGGTTGGAAGGCTTGGGGCAAGAGCATCATCTTTGCTCCAGAACTAGCTAACAACACCTTTGTCATCGACGGTCCTGAGACTGAGGAATGCCCAACGGCCAACCGAGCATGGAACAGTATGGAGGTAGAAGACTTTGGATTTGGATTGACAAGCACTCGCATGTTCCTGAGGATTCGGGAAACGAACACAACTGAATGCGACTCGAAGATCATAGGAACCGCCGTCAAGAACAACATGGCTGTGCATAGTGATCTGTCGTACTGGATAGAGAGCGGACTCAACGACACCTGGAAGCTTGANAGGGCNGTTCTAGGAGAAGTCAAATCATGCACCTGGCCAGAAACCCACACTTTGTGGGGTGATGGAGTTCTGGAAAGTGATCTCATCATACCCATCACCTTGGCAGGACCCAGAAGCAATCATAACAGGAGACCAGGGTACAAAACTCAGAACCAAGGTCCATGGGATGAGGGGCGTGTCGAGATTGACTTTGACTATTGCCCAGGAACAACAGTAACTATAAGTGANAGTTGCGGACACCGTGGACCTGCGGCACGCACAACCACTGAGAGTGGGAAGCTCATTACAGANTGGTGCTGCAGAAGCTGCACCCTCCCTCCACTGCGCTTCCAGACTGANAATGGCTGTTGGTANGGAATGGAAATTCGACCCACGCGGCACGACGAAAAGACCCTCGTGCAATCGAGAGTGAATGCATACAACGCCGACATGATTGATCCTTTTCAGTTGGGCCTTCTGGTCGTGTTCTTGGCCACCCAGGAGGTCCTTCGCAAGAGGTGGACGGCCAAGATCAGCATTCCAGCTATCATGCTTGCACTCCTAGTCCTAGTGTTTGGGGGTATTACGTACACTGATGTNCTGCGATATGTCATTCTCGTCGGCGCCGCGTTTGCTGAAGCAAACTCAGGAGGAGACGTTGTGCACTTGGCACTCATGGCNACATTCAAGATTCAACCAGTCTTTCTGGTGGCTTCCTTTTTGAAGGCAAGGTGGACCAACCAAGAGAGTATTTTGCTCATGCTTGCAGCTGCTTTCTTTCAAATGGCTTACTATGANGCCAAGAATGTTTTGTCATGGGAAGTGCCTGACGTTTTGAACTCTCTCTCCGTNGCNTGGATGATTCTCAGAGCTATAAGCTTCACCANCACCTCAAATGTGGTGGTGCCGCTGCTGGCCCTTCTGACACCTGGACTAAAATGCCTAAACCTTGATGTGTACAGAATTTTGCTACTCATGGTTGGAGTTGGAAGCCTCATCAAAGAAAAAAGGAGCTCTGCAGCAAAAAAGAAAGGGGCTTGCCTCATCTGCCTAGCGCTGGCGTCTACAGGAGTGTTCAATCCAATGATACTTGCAGCTGGGCTGATGGCTTGCGACCCCAACCGCAAGCGGGGCTGGCCTGCCACAGAAGTGATGACTGCAGTTGGACTCATGTTTGCCATCGTTGGGGGTCTGGCAGAACTTGACATAGACTCTATGGCTATCCCCATGACCATTGCCGGACTCATGTTCGTGGCATTTGTCATCTCTGGAAAGTCAACAGACATGTGGATTGAGAGGACGGCTGATATTACTTGGGAGAGTGACGCTGAAATCACAGGCTCTAGTGAAAGAGTAGATGTTAGGCTGGATGATGATGGAAATTTTCAATTGATAAATGACCCNGGGGCGCCATGGAAAATTTGGATGCTTAGGATGGCCTGCCTGGCAATAAGTGCCTACACACCTTGGGCGATTCTTCCCTCGGTCATTGGATTCTGGATAACCCTTCAGTACACAAAGAGAGGAGGTGTCCTTTGGGACACACCATCACCCAGGGAGTACAAGAAGGGTGATACCACCACTGGCGTTTACAGAATCATGACTCGAGGTCTGCTTGGCAGTTACCAAGCTGGAGCCGGAGTGATGGTAGAGGGAGTGTTCCACACACTATGGCACACCACTAAGGGAGCTGCTCTCATGAGTGGTGAAGGACGTCTGGATCCCTATTGGGGGAGCGTGAAAGAAGACCGGCTTTGCTATGGGGGGCCATGGAAACTCCAACATAAATGGAATGGACATGATGAGGTCCAAATGATTGTNGTGGAGCCAGGGNAAAATGTGAAAAACGTCCAGACTAAGCCCGGAGTGTTTAAGACACCAGAAGGAGAAATTGGGGCAGTTACGCTAGACTATCCCACTGGAACGTCAGGTTCCCCCATTGTGGATAAAAATGGAGATGTGATTGGATTGTATGGGAACGGCGTCATCATGCCTAATGGTTCATACATAAGCGCCATTGTGCAAGGAGAGAGAATGGAAGAACCGGCACCAGCTGGCTTCGAACCTGAAATGTTGAGGAAGAAACAGATCACTGTCCTTGATCTGCACCCCGGAGCAGGAAAGACGCGCAAGATACTTCCCCAAATCATCAAGGAGGCCATCAACAAAAGANTGAGGACGGCTGTACTGGCACCCACCAGGGTCGTTGCTGCTGAGATGTCTGAGGCCCTGAGAGGACTTCCCATCCGGTACCAAACCTCAGCAGTGCACAGNGAGCACAGTGGAAATGAGATCGTTGATGTCATGTGCCATGCCACTCTCACACACAGGCTGATGTCTCCACACAGAGTCCCCAATTACAATCTGTTCATAATGGATGAAGCCCATTTCACGGATCCAGCGAGCATCGCAGCCAGAGGATACATAGCAACCAAGGTTGAATTGGGCGAAGCCGCCGCGATTTTCATGACGGCAACGCCACCCGGGACTTCTGACCCCTTTCCAGAGTCCAATGCTCCTATCTCGGACATGCAAACAGAGATCCCAGACAGAGCCTGGAACACTGGATATGAATGGATAACTGAGTATGTTGGAAAGACCGTTTGGTTTGTTCCAAGTGTGAAAATGGGAAATGANATTGCCCTCTGTCTGCAACGGGCGGGGAAGAAGGTTATCCAGCTGAACAGAAAGTCCTATGAGACAGAGTACCCCAAGTGCAAGAACGATGATTGGGATTTTGTCATCACCACAGACATATCAGAAATGGGAGCAAACTTCAAGGCAAGCAGAGTGATCGACAGCCGCAAAAGCGTGAAACCCACCATCATTGAGGAAGGTGATGGAAGAGTCATNCTGGGGGAACCCTCAGCCATCACGGCTGCCAGCGCTGCTCAGCGGAGAGGACGCATAGGAAGAAACCCATCACAAGTTGGTGATGAGTATTGNTATGGAGGGCACACAAATGAGGATGATTCCAACTTTGCCCACTGGACAGAGGCTCGCATCATGCTAGACAACATCAACATGCCGAATGGTCTGGTGGCCCAACTATATCAGCCTGAGCGCGAGAAGGTGTACACCATGGACGGGGAATATAGGCTCAGAGGGGAAGAACGGAAGAACTTCCTTGAATTCCTGAGAACAGCTGATTTACCAGTCTGGCTCGCTTACAAAGTGGCAGCAGCAGGAATATCATACCATGACCGGAAGTGGTGCTTTGATGGACCTCGAACCAACACGATTCTTGAAGACAACAATGAAGTTGAAGTCATCACGAAGTTGGGTGAGAGAAAGATCNTAAGACCCAGGTGGGCGGATGCCAGAGTGTACTCAGACCACCAAGCTCTAANGTCCTTCAAAGATTTTGCATCAGGGAAACGATCACAAATCGGGCTCGTTGAGGTGCTCGGGAGAATGCCTGAGCACTTCATGGGGAAAACTTGGGAGGCGTTGGACACGATGTATGTGGTGGCAACCGCTGAAAAAGGAGGCCGAGCTCACAGGATGGCTCTTGAGGAGCTACCGGACGCCCTTCAGACAATAGCTTTGATTGCACTATTGAGTGTGATGTCCTTAGGTGTGTTNTTTCTACTCATGCAAAGGAAAGGCATTGGTAAGATTGGCTTGGGAGGAGTGATCTTAGGAGCTGCCACATTCTTCTGCTGGATGGCTGAAGTCCCAGGAACGAAAATAGCAGGCATGCTCCTGCTTTCCCTGCTGCTCATGATNGTTTTGATTCCGGAGCCGGAAAAGCAGCGCTCACAGACTGACAACCAGCTTGCCGTGTTCTTGATTTGTGTGCTCACACTGGTCAGCGCCGTGGCTGCCAATGAAATGGGTTGGCTGGACAAGACCAAGAATGACATNAGCAGCCTNTTGGGACACAAGCCAGAAACTAGAGAGACGACCCTGGGAGTTGAGAACTTTTTGCTTGATCTGCGGCCNGCTACAGCATGGTCGCTCTATGCCGTAACGACAGCCGTTCTTACCCCTTTGCTGAAACATCTAATCACGTCAGACTACATCAACACTTCGCTGACCTCAATAAACGTCCAAGCCAGTGCGTTGTTCACCTTGGCCAGAGGCTTCCCTTTTGTGGACGTTGGTGTGTCAGCTCTCTTGCTGGCGGCCGGGTGCTGGGGCCAAGTGACCCTGACTGTGACTGTGACTGCAGCTGCCCTGCTCTTTTGCCACTATGCTTACATGGTACCAGGCTGGCAAGCGGAAGCCATGCGATCCGCCCAGCGGCGGACAGCTGCTGGCATCATGAAAAATGCAGTGGTGGATGGGATCGTGGCTACTGATGTACCTGAACTTGAGCGAACAACTCCAGTCATGCAGAAAAAAGTTGGACAGATCATGCTGATCTTGGTGTCAATGGCCGCGGTTGTCGTCAATCCATCAGTGAGAACTGTCAGAGAGGCTGGAATTCTGACTACAGCAGCAGCAGTCACCTTATGGGAGAATGGTGCTAGTTCAGTGTGGAATGCAACGACAGCTATTGGGCTTTGTCACATCATGCGAGGAGGATGGCTCTCGTGTCTCTCCATCACGTGGACTCTCATCAAAAACATGGANAAACCAGGCCTCAAGAGGGGTGGAGCCAAGGGACGCACNCTAGGGGAAGTTTGGAAGGAGAGACTCAACCACATGACGAAGGAAGAATTTACCAGATACAGAAAAGAAGCCATCACTGAAGTCGACNGCTCCGCGGCAAAACATGCCAGGAGGGAGGGAAACATCACTGGAGGCCATCCAGTCTCACGGGGAACCGCGAAATTACGGTGGTTNGTGGAAAGGCGTTTCCTCGAGCCAGTGGGAAAGGTTGTGGATCTCGGATGTGGTAGAGGCGGCTGGTGTTATTACATGGCTACCCAGAAGAGGGTACAGGAAGTGAAAGGGTACACGAAAGGAGGACCTGGCCATGAAGAGCCACAACTGGTGCAGAGCTATGGTTGGAATATTGTTACCATGAAGAGCGGAGTCGACGTCTTCTACAGACCATCAGAAGCGAGCGACACACTGCTCTGTGACATTGGAGAGTCATCGTCAAGTGCCGAGGTAGAAGAACACCGCACCGTCCGTGTCTTGGAGATGGTGGAAGACTGGCTGCACAGGGGACCTAAGGAATTCTGCATCAAGGTGCTATGCCCTTACATGCCTAGAGTGATTGAGAAGATGGAAACACTCCAAAGGCGATATGGAGGTGGCCTTGTGAGAAACCCCCTTTCACGCAACTCTACCCATGAGATGTATTGGGTGAGCCACGCTTCAGGCAACATTGTCCACTCCGTGAACATGACAAGCCAGGTGCTTCTGGGGAGGATGGAAAAGAAAACATGGAAGGGACCCCAGTTTGAGGAAGATGTCAACTTGGGAAGTGGAACNNGGGCAGTAGGGAAGCCTCTCCTCAATTCTGATACTAGCAAGATCAAGANCCGAATTGAGAGGCTGAAGAAAGAATACAGCTCCACATGGCACCAGGANNCGAATCACCCCTACAGGACCTGGAACTACCACGGAAGCTATGAAGTGAAACCAACCGGCTCAGCCAGCTCCCTTGTGAATGGGGTAGTTAGATTACTNTCAAAACCATGGGACACTATCACCAATGTGACCACGATGGCCATGACAGACACCACTCCTTTCGGTCAACAACGAGTGTTCAAGGAAAAGGTGGANACAAAGGCTCCAGAGCCTCCAGAAGGGGTCAAATACGTCCTCAATGAGACCACGAACTGGCTGTGGGCTTTTCTAGCCCGCGATAAGAAACCCAGGATGTGTTCCCGGGAGGAATTCATTGGAAAAGTCAACAGTAATGCCGCCCTNGGAGCGATGTTTGAAGAACAGCACCTGGAAGAACGCCCGGGAAGCCGTGGAGGATCCAAAGTTTTGGGAGATGGTGGATGAGGAGCGTGAGGCGCATCTCCGTGGAGAATGCAACACCTGCATCTACAACATGATGGGAAAGAGAGAGAAGAAGCCTGGAGAGTTCGGCAAAGCTAAAGGCAGCAGAGCCATTTGGTTCATGTGGCTAGGGGCCCGCTTCCTGGAGTTTGAAGCTCTCGGATTCCTCAATGAAGACCACTGGCTGGGTAGGAAAAACTCAGGAGGAGGAGTTGAAGGCTTAGGACTGCAGAAGCTTGGGTACATCTTGAAGGAAGTCGGAACAAGGCCTGGAGGAAAGATCTACGCCGATGATACCGCAGGCTGGGACACACGCATCACCAAAGCTGACCTCGAGAACGAAGCGAAGGTTCTTGAACTGCTGGACGGAGAACACCGACGCCTAGCGCGGTCCATCATTGAGCTCACATACCGACACAAAGTCGTGAAAGTGATGAGGCCAGCGGCCGACGGGAAAACTGTGATGGATGTCATCTCCAGAGAGGATCAGAGAGGAAGTGGGCAGGTAGTGACTTACGCCCTGAACACCTTCACTAATCTAGCAGTTCAGCTGGTCAGAATGATGGAGGGGGAGGGGGTCATTGGACCTGACGATGTTGAAAAACTGGGAAAAGGAAAAGGCCCCAAGGTCAGAACCTGGCTGTTTGAGAATGGCGAGGAGCGTCTCAGTCGCATGGCCGTCAGCGGTGATGACTGCGTGGTGAAACCTTTGGACGACCGCTTCGCCACATCACTACACTTCCTAAATGCCATGTCAAAGGTCCGCAAAGACATCCAGGAATGGAAACCCTCGACAGGGTGGTATGACTGGCAGCAGGTCCCATTCTGTTCAAACCATTTCACGGAATTGATCATGAAGGACGGCAGGACGTTGGTGGTCCCGTGTCGTGGACAAGACGAGTTGATTGGACGGGCCAGAATCTCTCCAGGGGCTGGATGGAATGTGCGCGACACCGCCTGCCTGGCGAAGTCATACGCGCAGATGTGGCTGTTGCTTTATTTTCACCGTAGAGACCTGAGATTGATGGCTAACGCCATCTGTTCCGCTGTGCCTGTCAACTGGGTTCCCACAGGNCGTACCACCTGGTCGATCCACGCAAAAGGAGAATGGATGACGACAGAAGACATGCTCGCAGTCTGGAACAGAGTGTGGATTGAGGAGAATGAGTGGATGGAAGACAAAACACCAGTTGAGAGGTGGAGTGATGTTCCATACTCTGGAAAGAGAGAAGACATTTGGTGTGGCAGTTTGATTGGCACACGAACCCGCGCTACTTGGGCTGAAAATATCCATGTGGCAATCAATCAGGTCCGTTCNGTGATTGGAGAAGAGAAGTATGTGGATTACATGAGCTCNCTGAGGAGGTATGAAGACACCATTGTAGTTGAGGACACTGTTTTGTAAATAATAAAGCTGTATTGAGTAGTTGTANAGTTGTAGTGTTTATAGCAATTTGAATTATGATTAATTANTTAGGNTTAANATAGTATTATAGTTAGTTTAGTGTAAATAGGTTTTATTGAGAATGGAAGTCAGGCCAGATTAATGCTGCCACCGGAAGTTGAGTAGACGAGGTGCTGCCTGCGGCTCAACCCCAGGAGGACTGGGTGACCAAAGNTGCGAGGTGATCCACGTAAGCCCTCAGAACCGTCTCGGAAGGAGGACCCCACGTGCTTTAGCCTCAAAGCCCAGTGTCAGACCACACTCTAGTGTGCCACTCTGCGGAGAGTGCAGTCTGCGATAGTGCCCCAGGTGGACTGGGTTAACAAAGGCAAAACATCGCCCCACGCGGCCATAACCCTGGCTATGGTGTTAACCAGGGAGAAGGGACTAGAGGTTAGAGGAGACCCCGCGTCAAAAAGTGCACGGCCCAACTTGGCTGAAGCTGTAAGCCAAGGGAAGGACTAGAGGTTAGAGGAGACCCCGTGCCAAAAACACCAAAAGAAACAGCATATTGACACCTGGGATAGACTAGGGGATCTTCTGCTCTGCACAACCAGCCACACGGCACAGTGC
