# Supplementary material for: VirDetector: a bioinformatic pipeline for virus surveillance using nanopore sequencing
Source: Bioinformatics. 2025 Jan 21;41(2):btaf029. doi: 10.1093/bioinformatics/btaf029 (PMC11802467; doi:10.1093/bioinformatics/btaf029)
Supplement: btaf029_Supplementary_Data [file btaf029_supplementary_data.zip › a8752_supplementary_material.zip!/supplementary_material/Supplementary_Tables.docx]

**Table S1.** Programmes used in VirDetector.

| Nextflow | https://www.nextflow.io/ |
| --- | --- |
| POD5 | https://github.com/nanoporetech/pod5-file-format |
| Guppy | https://community.nanoporetech.com/docs/prepare/library_prep_protocols/Guppy-protocol/v/gpb_2003_v1_revax_14dec2018/guppy-software-overview |
| Dorado | https://github.com/nanoporetech/dorado |
| NanoComp | https://github.com/wdecoster/nanocomp |
| NanoFilt | https://github.com/wdecoster/nanofilt |
| Canu | https://github.com/marbl/canu |
| BLAST | https://blast.ncbi.nlm.nih.gov/doc/blast-help/ |
| Minimap2 | https://github.com/lh3/minimap2 |
| Samtools | https://www.htslib.org/ |
| Qualimap | http://qualimap.conesalab.org/ |
| MAFFT | https://mafft.cbrc.jp/alignment/server/index.html |
| Muscle | https://drive5.com/muscle5/ |
| CIAlign | https://github.com/KatyBrown/CIAlign |
| seqmagick | https://github.com/fhcrc/seqmagick |
| IQ-TREE2 | http://www.iqtree.org/ |
| MrBayes | https://nbisweden.github.io/MrBayes/ |
| FigTree | https://github.com/rambaut/figtree |
| Sniffles | https://github.com/fritzsedlazeck/Sniffles |
| KMA | https://bitbucket.org/genomicepidemiology/kma/src/master/ |
| Kraken2 | https://ccb.jhu.edu/software/kraken2/ |
| Krona | https://github.com/marbl/Krona |
| fpdf | https://pypi.org/project/fpdf/ |
| pypdf | https://pypi.org/project/pypdf/ |
| Biopython | https://biopython.org/ |

**Table S4.** Databases used in VirDetector.

| Viral reference sequences | https://ftp.ncbi.nlm.nih.gov/refseq/release/viral/viral.1.1.genomic.fna.gz |
| --- | --- |
| Viral Kraken2 | https://genome-idx.s3.amazonaws.com/kraken/k2_viral_20240605.tar.gz |
